# Reducing the GAP between science and clinic: lessons from academia and professional practice - part A: perceptual-auditory judgment of vocal quality, acoustic vocal signal analysis and voice self-assessment

**DOI:** 10.1590/2317-1782/20212021240en

**Published:** 2022-08-01

**Authors:** Mara Behlau, Anna Alice Almeida, Geová Amorim, Patrícia Balata, Sávio Bastos, Mauricéia Cassol, Ana Carolina Constantini, Claudia Eckley, Marina Englert, Ana Cristina Cortes Gama, Ingrid Gielow, Bruno Guimarães, Livia Ribeiro Lima, Leonardo Lopes, Glaucya Madazio, Felipe Moreti, Vanessa Mouffron, Katia Nemr, Priscila Oliveira, Marina Padovani, Vanessa Veis Ribeiro, Kelly Silverio, Thays Vaiano, Rosiane Yamasaki

**Affiliations:** 1 Centro de Estudos da Voz – CEV - São Paulo (SP), Brasil.; 2 Escola Paulista de Medicina – EPM, Universidade Federal de São Paulo – UNIFESP - São Paulo (SP), Brasil.; 3 Universidade Federal da Paraíba – UFPB - João Pessoa (PB), Brasil.; 4 Universidade Federal de Alagoas – UFAL - Maceió, AL, Brasil.; 5 APTA COMUNICAÇÃO - Recife (PE), Brasil.; 6 Universidade Federal de Pernambuco – UFPE - Recife (PE), Brasil.; 7 Centro de Fotobiomodulação e Saúde – CFOTOBIOS - Belém (PA), Brasil.; 8 Universidade Federal de Ciências da Saúde de Porto Alegre – UFCSPA - Porto Alegre (RS), Brasil.; 9 Universidade Estadual de Campinas – UNICAMP - Campinas (SP), Brasil.; 10 Faculdade de Ciências Médicas da Santa Casa de São Paulo – FCMSCSP - São Paulo (SP), Brasil.; 11 Universidade Federal de Minas Gerais – UFMG - Belo Horizonte (MG), Brasil.; 12 Clínica Bruno Guimarães Serviços de Fonoaudiologia e Fisioterapia - Fortaleza (CE), Brasil.; 13 Centro Universitário da Faculdade de Medicina do ABC – FMABC - Santo André (SP), Brasil.; 14 Complexo Hospitalar Municipal de São Bernardo do Campo – CHMSBC - São Bernardo do Campo (SP), Brasil.; 15 Universidade de São Paulo – USP - São Paulo (SP), Brasil.; 16 Faculdade de Odontologia de Bauru – FOB, Universidade de São Paulo – USP - Bauru (SP), Brasil.

**Keywords:** Voice, Voice Disorder, Voice Quality, Acoustics, Self-test

## Abstract

During the XXVIII Brazilian Congress of SBFa, 24 specialists met and, from a leading position on scientific research as a tool for connecting laboratory and clinic, five fronts of knowledge of the voice specialty were discussed as following: Perceptual-auditory judgment of vocal quality; 2. Acoustic analysis of the vocal signal; 3. Voice self-assessment; 4. Traditional techniques of therapy; 5. Modern techniques of electrostimulation and photobiomodulation (PBMT) in voice. Part “a” of this publication was associated with the consolidation of the analyses of the first three aspects. The trend in the perceptual-auditory judgment of vocal quality was related to the use of standard protocols. The acoustic evaluation of the vocal signal is accessible and can be done descriptively or by extraction of parameters, thus preferring multiparametric measures. Finally, the analysis of the individual himself closes this triad of voice documentation, which will be the basis for the conclusion of the evaluation, reference for monitoring progress, and evaluation of treatment results.

## INTRODUCTION

The field of study on voice is one of the five (5) original Specialist Registrations recognized by the General Medical Council in Brazil^(1)^, consisting of enough prolific production, both nationally and internationally acknowledged^(2)^. While the main role of science is the advancement of knowledge, not strictly limited to medical application, the clinical practice is responsible for offering the best treatment available to the individual, by taking into consideration the existing scientific evidence. Evidence-Based Clinical Practice (EBCP) will provide ratings on quality of evidence and strength of recommendations, establishing the prioritization steps, as well as the systematization and hierarchy of decision-making processes, in addition to considering patient values as a prerequisite for healthcare professionals' assessment. Nevertheless, quickly developed evidence that is representative of a specialty’s evolution is not always availabe and, moreover, adherence to scientific guidelines can vary, which requires an active dissemination and different strategies for implementation in clinical reality^(3)^. The distance between the research and the practice can be, in some cases, a gap and lead to an uncomfortable situation.

The clinical practice experience regarding voice studies is rather complex, seeing that it involves the use of established scientific literature, which is tangible, but less measurable aspects are also part of it, such as Metatherapy^(4)^, a professional competence based on clinical expertise constituting the “silent know-how” in vocal rehabilitation^(5)^.

The undergraduate courses, in Brazil or also abroad, tend to focus on integrating various theoretical contents in order to train speech-language pathologists, with a teaching structure that favors the association of theory with practice, relying on internship supervisors to provide students with guidelines for good clinical practices^(6)^. In consequence, graduate programs are not responsible for training specialists, due to several reasons, particularly the workload and the need for professional maturity

Similar to the workings of undergraduate internships, professional clinical care also integrates theoretical content into practice, but it is expected that the speech-language pathologist may be able to conduct individualized interventions, not only according to the individual's diagnosis, but taking into account the patient personality, and the context of speech interaction, especially in their social and professional roles. In clinic practice, decisions are made to solve the patient's problem, without having to force them into fitting a pre-existing theoretical model; in other words, there is a greater freedom than when compared to university clinics. There are rules to be respected, but there is also a deliberate flexibility acquired by professional background and competence.

Clinicians may use explicit knowledge, science development results and tacit knowledge, deriving from clinical experience and practice. Clinical questions must be structured and analyzed on a theoretical basis; however, a competent care support goes beyond this aspect and may involve innovative methodologies and approaches, even if not fully tested. Tacit knowledge is produced accordance with the respective amount of professional experience, developed skills and care value base, constituting another type of evidence that has a great impact on decision making. The faculty members' role is related to the encourage regarding the acquisition of theoretical references and explicit knowledge, all the while favoring the improvement of clinical reasoning and practical skills. Altough structured scientific knowledge is not enough for clinical development, it is an essential basis for a reasonable and accountable care support. Recognizing uncertainty in medical appointments is part of professional maturity and should not affect the speech-language pathologist integrity; whereas the risk of an uncritical learning that does not stimulate reflective thinking must never occur.

The present article aimed to record a summary of the Scientific Session that took place at the Mérito Mara Behlau room, on November 14th, 2020, as one of the activities of the XXVIII Brazilian Congress of SBFa. This research paper provided data on scientific methodology as instrumental to bring science and clinic together, establishing the field of study on voice as a constantly advancing science, demonstrating, side by side, both the explicit and the tacit theoretical bases regarding professional experience. Senior speech-language pathologists along with young scholars, from highly esteemed academic traditions, developed a dialogue with experienced clinicians respected by the market, concerning some approaches with different levels of evidence, among which, the perceptual-auditory judgment of vocal quality, the acoustic analysis of the vocal signal, voice self-assessment, as well as taking into consideration several aspects related to voice disorders, traditional techniques of therapy and, finally, modern techniques of electrostimulation and photobiomodulation applied to vocal rehabilitation. In the first part of this publication, essential considerations in the scientific quality of the researches were presented along with a commented analysis of the first three aspects discussed in this scientific session, namely: 1. Perceptual-auditory judgment of vocal quality; 2. Acoustic analysis of the vocal signal; 3. Voice self-assessment.

## SCIENTIFIC RESEARCH: FROM LABORATORY TO CLINICAL ROUTINE

The GAP between research and clinic is historical. Scientific researches were mainly based on expert opinions and case series from clinical practice. Most of these studies lacked validity, and their conclusions had no generalizability. As a consequence, the evidence described in the literature was often not applicable to practice, which distanced research and clinic. On the other hand, clinical practice was based on what was learned at graduation, and when faced with uncertainty, doubts were directed to specialist colleagues or references in the area^(7)^. Over time, scientific evidence has become increasingly easier for clinicians to access, with research papers made available by open access science journals, courses, congresses, books, scientific content lives, among others. Given the large volume of information available, the challenge that once was to be able to access it, became to be able to find good quality evidence with clinical applicability^(7,8)^.

**Figure 1:**
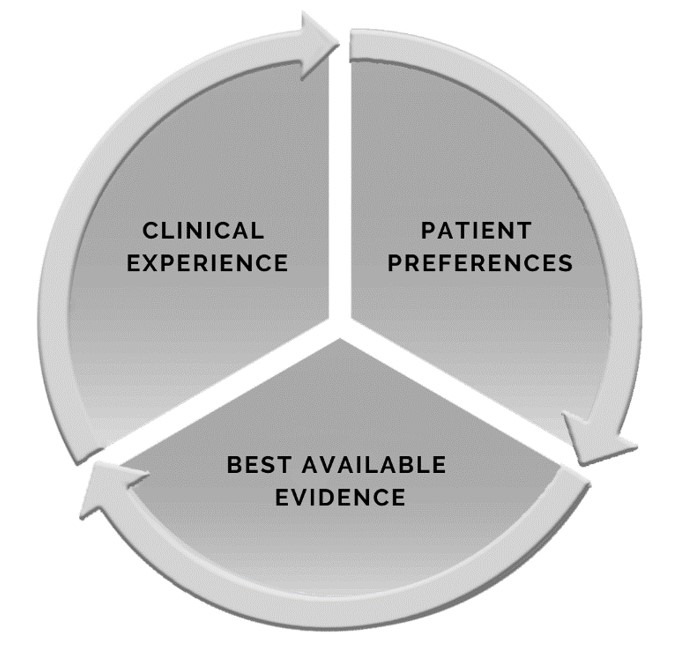
Components of Evidence-Based Practice

To reduce the GAP between science and practice, in the 1990s a movement called Evidence-Based Practice (EBP) emerged. EBP consists of associating the patient's perspectives and clinical experience, with the ability to consciously and judiciously analyze and apply the best scientific evidence available^(7-9)^ (Figure 1). This movement induced developing researches to gather evidences that could answer some of the doubts faced in practice. By using the EBP, it is possible to reduce uncertainty in clinical decision-making, to assist the choice of diagnostic procedures with greater accuracy, to curtail treatment time and cost, to reduce risk and increase the effect of treatment, among other benefits. This way, the clinical-epidemiological evidence can be integrated with the daily practical experience of each professional, that is, to offer ideal care support adaptive to real life conditions.

As a result of this movement, in 2004, the EBP in speech gained recognition, targeting voice disorders^(9)^. The implementation of EBP in clinical practice must comply with the following steps: to establish the clinical problem and develop a research question, for which the acronym PICO (Patient, Intervention, Comparison and Outcomes) or its variations can be used; to identify evidence that answers the databases' clinical question; to critically analyze the evidence (validity, reliability, quality and applicability); to apply scientific conclusions as the pillars for clinical practice, considering clinical experience as well as the patient's specificities and preferences^(9)^. In fact, the goal is to assist in the daily implementation of the EBP, and the association also recommends the acronym DECIDE, in which D stands for “Define the question”, E for “Extrapolate the evidence”, C for “Consider the experience”, I for “Incorporate the patient perspectives and needs”, D for “Develop a treatment plan” and E for “Examine your clinical decision”^(9)^.

However, the scientific vitality in Speech-Language Pathology (SLP) means that, during the trial phases, the clinician often finds a large volume of information with clinical heterogeneity and inconsistent quality of evidence. In view of this, the EBP proposes a hierarchical decision-making that prioritizes research by level of evidence, seeking to support the practice at the best level of evidence available. The traditional approach to hierarchy of evidence is to rank the level of collected data by the research designs that only considered the validation of medical evidences^(10)^. Having said that, after this primary classification, it is important that studies are reclassified acccording to the quality (or certainty) level of the evidence and the strength of the recommendations^(11)^.

Several types of research design are found in the literature, and an epidemiology based classification^(12)^, from *Clinical Trials* ^(13)^ and ASHA^(9)^, was chosen. Depending on the design, researches can be classified as primary data studies (individual studies where data is collected by the researcher from the source) and secondary data (combining findings from primary studies and providing conclusions to the body of evidence)^(9)^. Primary studies can be subdivided through comparability into observational (uncontrolled exposure and non-random allocation), quasi-experimental (controlled intervention and non-random allocation of participants to research groups) and experimental (controlled intervention and random allocation of participants in research groups). There is also a subgroup in quasi-experimental studies called before-and-after intervention studies (uncontrolled intervention, allocation cannot be random because there is only one intervention group). The observational category includes case-control studies (selection of groups based on the disorder, retrospective observation of exposure), cohort (selection of groups based on a common characteristic and classification based on exposure, prospective observation of the disorder) and cross-sectional (transversal analysis of the disorder with exposure and measurement contributions)^(12)^. Experimental studies (randomized clinical trials) can be subclassified according to phase, from adaptation to behavioral studies, as follows: Initial Phase I – laboratory studies; Phase I – analysis of the safety of immediate-effect intervention with nonpatients; Phase II – analysis of the safety and effectiveness of an immediate-effect intervention with patients; Phase III – analysis of safety and efficacy of intervention in patients; Phase IV – follow up and postmarketing^(13)^.

**Figure 2:**
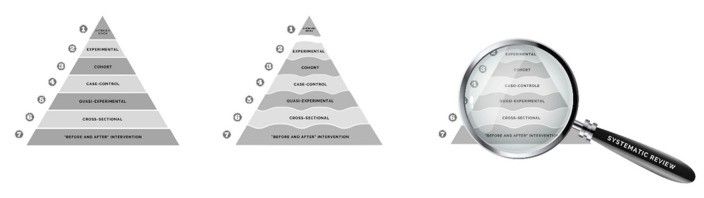
Evolution os the traditional pyramid to the proposed one, in which systematic revision is the framework for viewing and applying evidence

For the sake of ranking the level of evidence according to the design, it could be outlined a research proposal adaptation of the Oxford Center for Evidence-Based Medicine (EBM) and the ensuing revisions in design^(11)^ for speech-language pathology research. Indeed, it could be proposed that scientific research could be ordered as shown in Figure 2, depicting the evolution of the traditional evidence pyramid, from being based on the validity of the studies, to a pyramid with wavy lines, which highlights that the different types of design are not watertight categories, and as a result, it could be put forward a new proposal in which systematic revisions are framework through which evidence can be analyzed.

It is important to emphasize that the research design is directly related to the clinical question meant to be answered, and can be grouped as follows: diagnostic accuracy, prevalence and validation – cross-sectional studies; risk or protective factor and etiology – cohort or case-control studies; treatment and prevention – experimental, quasi-experimental study and before-and-after intervention^(9)^.

Along with the design, the study methodology must be taken into consideration, as to identify any limitations related to the evidence^(9)^. For this, instruments can be used to analyze the methodological quality or risk of bias. Secondary research with a systematic review design usually includes an assessment of methodological quality, as well as a single conclusion, which makes this design the one providing the best scientific support for clinical decision-making and for the development of healthcare guidelines^(14)^.

Currently, there is complementarity in EBP cycle between clinic and research. It is established in clinic through the doubts of practice, by researching the development of studies that may provide evidence to answer such doubts, circling back to clinic, where the evidence is comprehended and applied, bringing forth new doubts. The infinite cycle of partnership and complementarity is therefore sustained between the clinic and the research.

There are still many challenges for the EBP to be implemented and to meet the clinic's practical demands. Chart 1 shows some of these matters.

**Chart 1:** Challenges of implementing Evidence-Based Practice (EBP)

**Areas of the challenge**	**Challenges**
Search for Evidence	Limited and vague local terms
	Long period between development, submission, acceptance, and embargo for publication, which causes the availability of evidence to be delayed when compared to the speed of clinic practice
	Low level of evidence for research in some specific topics
Selection and comparison of evidence	Absence of sample calculation, small and heterogeneous samples
	Heterogeneous referral patterns and procedures related to voice disorders procedures
	Absence of standardization of outcome
	Diversity of dysphonia classifications
	Lack of follow-up to verify medium and long-term effects
	Mutability of temporal variables in the intervention
Evidence intake	Low consumption of studies by clinicians
	National health plans do not demand a scientific basis for the practice

One of the great issues to be faced in the future is implementation science. Only this way will it be possible to offer the best speech therapy care for patients with voice disorders.

### Perceptual-auditory judgment of vocal quality

The perceptual-auditory judgment of vocal quality is commonly called Auditory-Perceptual Assessment (APA) of voice and is the main instrument of speech-language pathology vocal assessment. In this form of analysis, the perceptual-auditory judgment to approach responses to sounds by stimulus was systematicallly used^(15-19)^. The fact that the voice is a perceptual phenomenon by nature makes the perceptual analysis the best evaluation strategy for voice disorders, being considered the gold standard of voice assessment. Perceptual descriptions are intuitive and clinically meaningful^(15-18)^.

Through the auditory-perceptual judgment, it is possible to: describe the patient's vocal characteristics, identify whether the voice is normal or deviated, classify the predominant type of deviation (rough, breathy, tense or a combination) and the intensity of the alteration^(18)^; verify vocal functionality using tasks that test different adjustments of the laryngeal and perilaryngeal muscles^(20)^; assist in the differential diagnosis of dysarthria, including flaccid, spastic, ataxic, hypokinetic, hyperkinetic and mixed^(21)^; compare results of pre- and post-intervention treatments, as to medication, surgery or vocal rehabilitation; and to elaborate the clinical reasoning for the vocal disorder assessment, for the decision-making and for the vocal rehabilitation process^(17,18,20-22)^.

The subjectivity involved in this type of assessment is one of the main criticisms aimed at perceptual analysis. There are factors that may interfere in the result of the perceptual analysis. Some of these factors involve random errors, which are difficult to control. Others involve systematic errors, known errors that are more manageable, such as auditory processing training, the evaluation scale or test protocol, vocal parameters and speech tasks. Auditory training is essential for both the development of internal references to little experienced evaluators, and for the recalibration of affordance perception to experienced evaluators^(15)^. It can be performed using human voices, with different types and degrees of vocal deviations, as well as synthesized voices. The use of anchor stimuli offers external references to the patients and calibrates the auditory perception^(19,23)^.

There are different standardized protocols for perceptual analysis. The Japanese GRBAS scale^(24)^ and the CAPE-V25 Protocol^(25)^ are widely used for clinical and scientific purposes. Both contain highly reliable vocal parameters, such as G - general degree of vocal deviation, roughness and breathiness^(17,23,26)^. While the GRBAS uses a 4-point numerical scale, the CAPE-V protocol uses the 100mm visual-analog scale, which offers greater precision in the analysis, allowing a more accurate assessment of vocal progress in therapy, even if in a lower magnitude. Both protocols have a limited number of parameters to be evaluated, which reflects the current trend in the literature; however, CAPE-V provides for the recording of voice types less commonly seen in clinic.

The choice of speech tasks is one of APA's key points. There are less specific speech tasks, such as sustained vowel emission, counting numbers, spontaneous conversation and singing voice emission, such as the “happy birthday” song; there are also more specific tasks, to test frequency variation (usual, high, low and glissando) and intensity (usual, weak and strong) of the Vocal Dynamic Field (VDF), which provide information on the functionality of the laryngeal and perilaryngeal muscles^(20)^; the use of phonetically motivated phrases, such as those from CAPE-V, helpful for differentiating neurological dysphonia from behavioral dysphonia; finally, laryngeal and phonoarticulatory diadochokinesia tests become important in dysphonia due to altered motorcontrol^(21,27)^. Vowels tend to emphasize the sound source characteristics and the connected speech samples allow a more comprehensive assessment of voice use, with aspects that can easily be minimized if only sustained vowel emissions are considered.

In addition to the abovementioned factors that may interfere with the APA, studies show that cognitive biases, such as knowledge of clinical history, diagnostic information and clinical context, can influence APA^(28-30)^. Consequently, researchers and clinicians should be aware of the possibilities of automatic categorization triggered by available knowledge and previous experience in performing APA.

### Acoustic analysis of the vocal signal

Another important instrument of vocal assessment, which combined with the perceptual-auditory judgment is of great clinical value, is the acoustic analysis of the vocal signal. This analysis, considered to be less subjective than the perceptual-auditory judgment, has the following main objectives: to document voice quality; to quantify several aspects of voice production; to detect vocal and/or laryngeal disorders; to perform trials; to monitor the therapy process and to acknowledgepatient voice^(31,32)^. Furthermore, it plays an important role in helping the evaluator to actually see what is being heard^(33,34)^.

Historically, acoustic assessment was restricted to large laboratories and university research centers due to the need to use expensive computers (minicomputers) with high processing capacity^(31,32)^. The analysis was essentially carried out in the sustained vowel, as it varies less than the connected speech, allowing for an easier parameter extraction^(35-37)^. There were major difficulties envolved in analyzing voices with large vocal deviations due to the need to identify the individual limits of each cycle, which is less evident in very ? voices^(38)^. In recent decades, technological advances have allowed acoustic analysis to be used in multiple free softwares foi Personal Computers (such as Praat, by Paul Boersma and David Weenink, Institute of Phonetic Sciences, Amsterdam) or even low cost softwares (such as VoxMetria and FonoView, CTS Informatica, Brazil), broadening its use, and inevitably, related criticism and considerations^(39)^.

Acoustic analysis, although often associated with parameter extraction, can also be descriptive, essentially based on spectrography, a qualitative analysis of the vocal signal that allows the evaluation of any types of vocal signals, even in voices with extreme deviations, both from samples of sustained vowel and from speech^(40,41)^. The analysis of a voice acoustic spectrography requires a trained professional and is more difficult to be standardized, since the trace analysis is a perceptual-visual judgment with a good dose of subjectivity. In spite of that, research has been carried out towards developing a standard protocol for this analysis^(42-44)^ that is so greatly relevant to the clinic because of its immediate visual feedback contribution to the clinician and to the patient.

Assessment by the acoustic parameter, on the other hand, is easy to standardize, since the analysis provides a number to work with. Traditionally, parametric analysis is performed by stand-alone measures, such as: fundamental frequency, robust measure of scale; short-term perturbation measures, such as jitter and shimmer; noise measures, such as Signal to Noise Ratio (SNR), Harmonic to Noise Ratio (HNR), Glottal to Noise Excitation (GNE), among others. It is worth mentioning the referred research seeks to relate acoustic measures with the auditory-perceived vocal quality and the intensity of dysphonia^(36)^, but alterations in isolated acoustic parameters often do not reflect what the clinician hears in practice. Such parameters generally consider the sustained vowel as a task for analysis, which makes the assessment less assertive and less representative of the voice as a whole^(37)^. Hence, there is a search for measures that are more reliable and that also consider connected speech, such as the *Cepstral Peak Prominence* (CPP)^(37,45,46)^. The use of multiparametric measures, recently introduced in the literature, has become very popular, such as the *Acoustic Voice Quality Index* (AVQI)^(37)^ and the *Acoustic Breathiness Index* (ABI)^(16)^, both validated for Brazilian Portuguese^(47,48)^ and extracted by a script from the Praat program, free of charge. It is possible that one of the main challenges for the use of these measures in the clinical routine may be the lack of training in extraction programs, since Praat is not so user-friendly for application in the vocal clinic practice.

Perceptual-auditory judgment and acoustic analysis can be considered as two sides of the same coin, being regarded as the foundation of voice assessment from the clinician's point of view. Both must be carried out with great care, requiring training on the part of the evaluators and the selection of reliable parameters. While the perceptual-auditory judgment favors the description of the voice quality general impressions, the acoustic analysis allows the identification of essential aspects of the source contribution, along with the resonance filters in voice production.

### Voice self-assessment protocols

Self-assessment in the Brazilian voice clinic began a little over a decade ago with the validation of the Voice-Related Quality of Life (V-RQOL) protocol^(49)^. Voice is biologically defined and socially shaped^(50)^. It has multidimensional attributes that demand analysis from different perspectives during the diagnostic process of dysphonia. The clinician has the expertise, but it is the patient who has the experience of voice alteration, and the same problem when evaluated from different points of view will not always be equal to different evaluators. Patients who report different perceptions provide more information than evaluators can mutually agree upon^(51)^.

Self-assessment helps in understanding the difficulties experienced by the patient^(52,53)^. It is an important resource for clinical monitoring and can be a significant asset in diagnosis^(54)^. There are self-assessment instruments that may help in screening and diagnosis, in all cases accuracy and precision are essential^(55)^. To track a vocal disorder, the instrument must have high sensitivity, identifying people who have a voice alteration or present risk factors for dysphonia. As for the diagnosis, the instrument must have high specificity, as it cannot capture other alterations/disorders besides dysphonia.

Currently, in Brazil, the following protocols are available in the voice clinic practice: Voice-Related Quality of Life (VRQOL)^(49)^; Voice Handicap Index (VHI)^(56)^; URICA-VOICE Scale (URICA-VOICE)^(57)^; Vocal Tract Discomfort Scale (VTDS)^(58)^; Voice Activity and Participation Profile (VAPP)^(59)^; Vocal Symptom Scale (VoiSS)^(60)^; Dysphonia Coping Strategy Protocol (PEED)^(61)^; and the Vocal Fatigue Index (VFI)^(62)^. In addition to others for more specific populations, such as the Modern Singing Handicap Index (MSHI)^(63)^, Classical Singing Handicap Index (CSHI)^(64)^, pediatric Voice-Related Quality of Life (pVRQOL)^(65)^, Transsexual Voice Questionnaire Male to Female (TVQ^MtF^)^(66)^, Screening for Voice Disorders in Older Adults (RAVI)^(67)^ and the Pediatric Vocal Symptoms Questionnaire (PVSQ)^(68)^, among others.

By means of the protocol application, it is possible to measure the impact of the disorder on the patient's social, personal and professional relationships, to differentiate or group patients, to predict individual results, to monitor patient's evolution, to assess the effectiveness of the therapy and to prioritize problems in the intervention process, as well as helping in the decision-making concerning therapeutic discharge^(60,65,69)^. The clinician must then identify what they want to map in order to choose the most suitable protocol(s) for the patient.

It is important that speech-language pathologists try to prioritize, in their clinical routine, the use of validated scientific research instruments. Validation is an evaluation process that uses tests with results interpretation that are valid (providing proposal measurements), reliable/accurate (with test-retest repeatability) and unbiased (with information impartiality)^(67)^.

There are several guidelines to develop instrument validation processes, traditionally carried out by different areas, with emphasis on Education and Psychology^(67,70-72)^. Therefore, it is essential that the researcher define a line of validation and strictly follow all the steps recommended by the authors in order to take measurements: 1. Validity of evidence may be achieved through test content, response processes, internal consistency, and relationship between variables; 2. Reliability/Accuracy; 3. Unbiased test; 4. Accuracy; and 5. Evidence of validity based on testing consequences^(67,72)^.

Beside the levels of validity evidence, the researcher must choose the philosophical movement that will guide the test validation. In this standpoint, we could highlight a review that evaluated nine self-assessment instruments concerning V-RQOL^(73)^. After a careful analysis, the authors reported that the questionnaires most commonly used in the area of voice presented developmental problems and deficits in some of their psychometric properties. Concluding by suggesting the implementation of more contemporary methods for the validation and evaluation of psychometric measures.

All voice instruments in Brazil were validated based on the Classical Test Theory (CTT), internationally, there are already efforts in the development of studies to validate questionnaires based on more current models, such as the Item Response Theory (IRT)^(74-77)^. Within this approach, it is worth briefly mentioning the differences between these theories.

The CTT advocates for a structure in which the analysis depends on the collected sample; assigning a single value to the items, thus obtaining a full score from the simple sum of the items; the same number of marked questions (which may vary), receive the same score value; and, consequently, does not differentiate patients in terms of attribute aptitude. The IRT seeks to fill the gaps presented by the CTT, since its generalization is independent of test and sample; the items are established as a basic unit, therefore, each one will have its part in influencing the latent trait, deriving from its difficulty and discrimination parameters. For this reason, the instruments validated by IRT do differentiate individuals according to the level of aptitude/commitment tested, in order to improve the accuracy of the instrument. That being said, a larger sample is needed along with more variability in problem severity, together with more sophisticated statistical models and softwares^(78-80)^.

An interdisciplinary and multi centered group was founded in Brazil, focused in developing the stages of validity evidence related to self-assessment protocols in voice, using the IRT. So far, seven instruments have been validated, namely: Vocal Tract Discomfort Scale^(81,82)^; URICA-Voice Validated (URICA-VV)^(83,84)^; V-RQOL^(85)^; Voice Symptom Scale (VoiSS)^(86)^; Dysphonia Coping Strategy Protocol^(86)^; Voice Handicap Index^(87)^; and Perceived Present Control Intervention for Voice (CPP-V)^(88)^.

Most of these protocols, validated through IRT, emphasized the difficulty patients had in understanding the difference between intensity and frequency, as well as answering the Likert scales (type); all items had the parameters calibrated in conformity with the individual level of information; measures regarding sensitivity, specificity and accuracy of the instruments were satisfactory; in addition to all the instruments mentioned, there is a new calculation methodology, based on IRT parameters, with the possibility of a new cutoff scores. On that account, all these new protocols followed the contemporary trend by simplifying research instruments in the area of Psychometrics, either with the reduction of items, with an unifactorial structure and/or with dichotomous responses; which allows for the classification of subjects with indicatives of vocal risk.

Such collective effort to produce new scientific knowledge could be reflected in clinical practice, for a better understanding of the genesis and processes that surround dysphonia, such as: instruments able to detect dysphonia even at an early stage, since most of the impacts on quality of life are daily life limitations adress physical issues and physical symptoms, rather than emotional ones; and those symptoms may or may not have show signs of phonotraumatic injury. Finally, one should know more about the patient's cognitive and behavioral aspects to make use of coping strategies, perceived present control and the patient's stage of readiness, as to encourage a more active patient and a more assertive therapist in vocal rehabilitation. Thus, it is believed that advances in scientific research with the use of IRT in the validation of vocal self-assessment instruments will facilitate the clinical routine of the speech-language pathologist.

## COMMENTS

Voice quality is an essential aspect in the assessment of a patient with a voice complaint, whether in the context of screening, diagnostic confirmation or monitoring the treatment of a voice disorder. Perceptual-auditory judgment and acoustic analysis are the main clinical tools to characterize and classify voice quality. Both assessment modalities must be seen as interdependent strategies and must be interpreted as integrated with the clinical history of the voice disorder, the self-assessment of aspects related to the voice issues in the individual's life and the laryngeal medical examination.

The speech-language pathologist must be able to collect auditory and acoustic information from the patient, synthesize it, interpret it and make a decision about the best intervention applicable to the case, or judge the effectiveness of an already implemented approach^(89)^. One of the great challenges in the voice field of study is to understand the role of the perceptual-auditory judgment and the measurements obtained by the acoustic analysis in the decision-making process. Although, traditionally, there may be a consensus among clinicians that vocal assessment is multidimensional, there is no scientific evidence to support this statement^(90)^.

Specifically concerning the perceptual-auditory judgment, one of the goals is to improve the reliability and accuracy in the voice quality characterization. As a general rule, listeners with similar auditory experiences or who had auditory training during their education tend to have better inter and intra-individual reliability^(34)^. Along these lines, two important points must be considered: the prior training of evaluators to improve the reliability and accuracy in the perceptual-auditory judgment; and the establishment of basic specifications for this training, so that the analyzis performance can be effectively improved^(15,91,92)^. Training should involve skills associated with central auditory processing and specific aspects related to perception tasks. Unexperienced evaluators showed less reliability in judging the general degree of vocal deviation when confronted with limitations in temporal resolution skills and binaural interaction^(93)^. Another study with tuned and untuned listeners showed that the evaluator's vocal tuning is not a prerequisite for performing a good perceptual-auditory voice assessment. Whereas participants with difficulty in processing temporal patterns had lower intra-subject reliability in the perceptual-auditory judgment, regardless of whether they were in tune or not^(94)^. Such findings reinforces the importance of training auditory skills in programs, in order to develop the ability to perform the perceptual-auditory judgment of voice quality.

Still, the greatest contribution of acoustic analysis is to enable the vocal signal's documentation, the monitoring of this signal throughout the rehabilitation or vocal improvement and the possibility of quantifying the auditory perceived deviation, as well as visually characterizing it in the spectrography. There are 15 models referred to in the literature for voice acoustic characterization^(95)^ and countless measurements that can be obtained from these models. Therefore, the clinician needs to decide which measures are most effective for specific purposes in the clinical context.

The search for acoustic measures that represent certain vocal quality descriptors, such as roughness and breathiness, is an old challenge for the scientific community. The large amount of acoustic measures available with the objective of correlating the acoustic findings with the perceptual-auditory ones was evidenced in a meta-analysis that evaluated acoustic measures used to assess sustained vowels and connected speech^(16)^. The authors found 85 acoustic measures for breathiness and 86 measures for breathy voice quality. Since meta-analysis is a type of literature review in which different studies are compared using statistical methods that reduce bias from methodological differences among them, this type of study has a high level of evidence. After analyzing the selected studies, the authors identified 12 measures with potential for correlation with breathiness and 14 for roughness. One noteworthy aspect found is that the choice of acoustic measures to be used by the clinician or researcher must be guided by the purpose of the intended analysis.

Discrimination between normal and deviated voices by means of acoustic measurements is another difficulty for researchers in the area and, therefore, it was the research objective of an important study that analyzed a database with 482 voices^(41)^. Different measures were compared individually and in combination to assess performance in discriminating voices. The differential of this study is the use of traditional, cepstral, non-linear measures, and recurrence quantification analysis. The results indicated that Cepstral Peak Prominence-Smoothed (CPPS) is the most accurate acoustic measure to discriminate normal from altered voices and that combined measurements (traditional, such as Glottal to Noise Excitation (GNE) and recurrence quantification analysis (entropy - ENTR) that may have promising results in voice discrimination. In view of this, CPPS is established as the most robust measure, both for verifying the correlation with breathiness^(16,41)^ and for the discrimination of normal and altered voices.

An important aspect to be highlighted in the acoustic analysis and in the perceptual-auditory judgment is the speech tasks in use. They must allow access to vocal functionality in terms of frequency variation (low, high and the alternation between them), intensity (weak, habitual and strong)^(96)^, as well as resistance (emission in maximum phonation time at different intensities and frequencies) and laryngeal efficiency (laryngeal diadochokinesia)^(97)^. At the same time, the Sound Pressure Level (SPL) must be a controlled variable and recorded at the time of voice data collection. Admittedly, voices produced with SPL below 70 or above 80dB in an acoustically treated clinical environment can distort the vocal deviation perception, under or overestimating the acoustic and perceptual characteristics of the voice^(98)^.

Therefore, the recommendation is that the perceptual-auditory and acoustic analysis of vocal quality must involve controlled speech material and, at least in hard cases, tasks with sustained vowel emission in weak, comfortable and strong intensity, since the quantitative differences and qualitative characteristics of these emissions can help to understand the laryngeal dynamics, especially between vocal hyperfunctioning conditions with or without tissue damage^(96)^. Connected speech tasks can also be evaluated under various emission conditions.

If, on the one hand, perceptual-auditory judgment and acoustic analysis are the main tools of semiology from the clinician's perspective, the self-assessment of the individual with voice problems, developed more intensively in the last two decades, has brought great advances in the comprehensive understanding of what does it mean to live with dysphonia. The inclusion of the patient's point of view in the assessment is a mandatory part of the vocal clinic routine. There is a huge range of voice self-assessment protocols already validated in Brazil, with different perspectives of analysis components, such as quality of life^(49,56,59,65)^ pathophysiology and presence of symptoms^(18,59,62,68)^, as well as behavior and cognition^(57,61)^. The question that always arises for clinicians, especially for beginners, is: “which vocal self-assessment protocol should I use with my patient?” That is why clinical reasoning is so important, with acknowledgement of the vocal complaint, sampling anamnesis data and guiding the decision-making process axis for patient evaluation, with the possibility of choosing more than one questionnaire.

The validation of voice self-assessment protocols in Brazil by the Classical Test Theory (CTT) lasted for over two decades, beginning with the VRQOL^(49)^. At that point, an important line of research for the validation of voice self-assessment protocols emerged in Brazil, with studies carried out at different academic levels, from specialization to doctoral studies. A recent publication compiled several recommendations regarding validation of tests in Speech-Language Pathology^(67)^, being one of the most cited articles in the CoDAS journal in the 2017-2020 quadrennium, proving that there the validation field is interesting to speech-language pathologists, mainly because validated protocols give greater security to the clinician's decision-making process.

After these initial advances, it became visible that there was a demand for further studies contemplating self-assessment instruments as a research object, as to ensure that they may evolve into increasingly efficient tools, safely reflecting the concerns presented by patients^(73)^. More recent lines of research have been dedicated to a deeper investigation of the psychometric structure related to vocal self-assessment instruments, aiming to better understand their potential and deficiencies, in addition to further reinforcing their validity and reliability. Preliminary studies in this area^(84,85,99)^ pointed out the ability to differentiate between items of the same instrument, leading to the conclusion that such items must be considered differently in order to obtain the total instrument score and their interpretation.

Such reflections culminated in the application of more contemporary validation theories for these instruments, such as the Item Response Theory (IRT), designed to solve such deficiencies by assigning different weights to each investigated item, in line with its influence towards the investigated parameter. The progress of these researches has already resulted in new, updated and psychometrically more robust versions of several instruments of vocal self-assessment, which have direct implications for the better quality of information obtained by these instruments, both in the clinical and academic scope. However, seeing that the resulting scores are not easy to be obtained or be clinically interpreted, their use is not quite widespread in clinical routine, as of yet.

The teaching-learning process of the perceptual judgments, acoustic and voice self-assessment protocols analysis can only be carried out properly if it is integrated with a clinical reasoning program since the beginning of speech-language pathologists' academic training. The data collected must be meaningfull and be part of the clinical decision-making. Clinical reasoning, which is based on a good anamnesis and an accurate patient assessment, is a cognitive skill to be developed through experience or training^(100)^. To develop clinical reasoning should be a key concern in the training of young speech-language pathologists, and can be promoted by a theoretical and structured approach, associated with the observation of experienced clinicians in practice at universities. This process empowers the professional, grants autonomy and promotes essential skills for interdependent work in the healthcare area.

## CONCLUSION

The field of study on voice is quite agile and has undergone a constant and intense renovation in the last two decades. Vocal functionality and conscious clinical decision should be the speech-language pathologists' main approach towards the patient. The perceptual-auditory analysis is of fundamental importance as a “voice spokesperson”, translating the individual's vocal identity into perceptual terms. Acoustic analysis, combined with auditory analysis, is the most trustworthy vocal documentation, providing the description of the source-filter contribution and resonant components of speech sound production, as well as allowing for the recording of the individual's entire communication style. The clinician has expertise in the area, but it is the patient who lives with the voice disorder, and it is only through self-assessment protocols that this experience can be translated into how it impacts on the family, social, professional and emotional aspects of life. Science has simplified this clinical assessment of the patient's point of view, with the revalidation of self-assessment protocols regarding the impact of voice problems on different aspects of life; today, it is understood that all the investigated items have different relevance, and the knowledge that this information has already generated will inspire the development of programs aimed at attention and prevention of voice disorders based on scientific data. The patient’s essential documentation comprises three analyses that allow for the treatment results’ evaluation: auditory, acoustic and self-assessment of the disorder impacts.

## References

[1] Brasil (1996). Resolução nº 157, de 13 de abril de 1996.

[2] Behlau M, Carroll L (2021). Vocal rehabilitation or voice therapy at Journal of Voice: a 30-year analysis on publications.

[3] Murad MH (2017). Clinical Practice Guidelines: a primer on development and dissemination. Mayo Clin Proc.

[4] Helou L (2017). Crafting the dialogue: meta-therapy in transgender voice and communication training. ASHA Perspectives..

[5] Iwarsson J (2015). Reflections on clinical expertise and silent know-how in voice therapy. Logoped Phoniatr Vocol.

[6] Fernandes FD, Wertzner HF (2014). Competence-based curricula for the education of speech-language pathologists and audiologists in Brazil. Folia Phoniatr Logop.

[7] Kara-Junior N (2014). Medicina baseada em evidências. Rev Bras Oftalmol.

[8] Lopes LW, Moreti F, Ribeiro LL, Pereira EC (2019). Fundamentos e atualidades em voz clínica..

[9] ASHA: American Speech and Hearing Association (2020). Evidence-Based Practice (EBP).

[10] Group GW (2006). Grading of Recommendations Assessment, Development and Evaluation (GRADE).

[11] Murad MH, Asi N, Alsawas M, Alahdab F (2016). New evidence pyramid. BMJ Evid Based Med.

[12] Medronho RA (2006). Epidemiologia..

[13] Clinical Trials (2021). Glossary of common site terms.

[14] Miranda VSG, Marcolino MAZ, Rech RS, Barbosa LR, Fischer GB (2019). Fonoaudiologia baseada em evidências: o papel das revisões sistemáticas. CoDAS.

[15] Oates J (2009). Auditory-perceptual evaluation of disordered voice quality: pros, cons and future directions. Folia Phoniatr Logop.

[16] Barsties B, De Bodt M (2015). Assessment of voice quality: current state-of-the-art. Auris Nasus Larynx.

[17] Iwarsson J, Sundberg J (1998). Effects of lung volume on vertical larynx position during phonation. J Voice.

[18] Behlau M (2019). The 2016 G. Paul Moore Lecture: Lessons in Voice Rehabilitation: Journal of Voice and Clinical Practice. J Voice.

[19] Santos PCM, Vieira MN, Sansão JPH, Gama ACC (2019). Effect of auditory-perceptual training with natural voice anchors on vocal quality evaluation. J Voice.

[20] Behlau M, Madazio G, Feijó D, Pontes P, Behlau M (2001). O livro do especialista..

[21] Duffy JR (2020). Motor speech disorders: substrates, differential diagnosis, and management..

[22] Yamasaki R, Gama ACC, Lopes L, Moreti F, Ribeiro L, Pereira EC (2019). Fundamentos e atualidades em voz clínica..

[23] Brinca L, Batista AP, Tavares AI, Pinto PN, Araújo L (2015). The effect of anchors and training on the reliability of voice quality ratings for different types of speech stimuli. J Voice.

[24] Hirano M (1981). Clinical examination of voice..

[25] Kempster GB, Gerratt BR, Verdolini Abbott K, Barkmeier-Kraemer J, Hillman RE (2009). Consensus auditory-perceptual evaluation of voice: development of a standardized clinical protocol. Am J Speech Lang Pathol.

[26] Freitas SV, Pestana PM, Almeida V, Ferreira A (2014). Audio-perceptual evaluation of Portuguese voice disorders - an inter - and intrajudge reliability study. J Voice.

[27] Padovani M, Diaferia G, Lopes LW, Moreti F, Ribeiro LL, Pereira EC (2019). Fundamentos e atualidades em voz clínica..

[28] Solomon NP, Helou LB, Stojadinovic A (2011). Clinical versus laboratory ratings of voice using the CAPE-V. J Voice.

[29] Eadie T, Sroka A, Wright DR, Merati A (2011). Does knowledge of medical diagnosis bias auditory-perceptual judgments of dysphonia?. J Voice.

[30] Costa FP, Yamasaki R, Behlau M (2014). Influence of clinical context in characterization of severity of vocal deviation. Audiol Commun Res.

[31] Baken RJ, Baken RJ (1987). Clinical measurement of speech and voice..

[32] Bodt M, Heylen L (2014). Stemstoornissen: hanboek voor de kliniche paktijk..

[33] Chan KM, Yiu EM (2002). The effects of anchors and training on the reliability of perceptual voice evaluation. J Speech Lang Hear Res.

[34] Bele I (2005). Reliability in perceptual analysis of voice quality. J Voice.

[35] Askenfelt AG, Hammarberg B (1986). Speech waveform perturbation analysis: a perceptual-acoustical comparison of seven measures. J Speech Hear Res.

[36] Maryn Y, Roy N, De Bodt M, van Cauwenberge P, Corthals P (2009). Acoustic measurement of overall voice quality: a meta-analysis. J Acoust Soc Am.

[37] Maryn Y, Corthals P, van Cauwenberge P, Roy N, De Bodt M (2010). Toward improved ecological validity in the acoustic measurement of overall voice quality: combining continuous speech and sustained vowels. J Voice.

[38] Titze IR (1995). Workshop on acoustic voice analysis: summary statement..

[39] Brockmann M, Drinnan MJ, Storck C, Carding PN (2011). Reliable jitter and shimmer measurements in voice clinics: the relevance of vowel, gender, vocal intensity, and fundamental frequency effects in a typical clinical task. J Voice.

[40] Baken RJ, Baken RJ (1987). Clinical measurement of speech and voice..

[41] Lopes L, Vieira V, Behlau M (2022). Performance of different acoustic measures to discriminate individuals with and without voice disorders. J Voice.

[42] Lopes LW, Alves GAS, Melo ML (2017). Content evidence of a spectrographic analysis protocol. Rev CEFAC.

[43] Lopes LW, Silva ACF, Silva IM, Paiva MAA, Silva SIDN, Almeida LNA (2022). Evidence of internal consistency in the spectrographic analysis protocol. J Voice.

[44] Bastilha GR, Pagliarin KC, Moraes DAO, Cielo CA (2021). Spectrographic Vocal Assessment Protocol (SVAP): reliability and criterion validity. J Voice.

[45] Watts CR, Awan SN, Maryn Y (2017). A comparison of Cepstral Peak Prominence Measures From Two Acoustic Analysis Program. J Voice.

[46] Patel RR, Awan SN, Barkmeier-Kraemer J, Courey M, Deliyski D, Eadie T (2018). Recommended protocols for instrumental assessment of voice: american Speech-Language-Hearing Association expert panel to develop a protocol for instrumental assessment of vocal function. Am J Speech Lang Pathol.

[47] Englert M, von Latoszek BB, Maryn Y, Behlau M (2021). Validation of the Acoustic Voice Quality Index, Version 03.01, to the Brazilian Portuguese Language. J Voice.

[48] Englert M (2020). Validação do Acoustic Voice Quality Index (AVQI) e do Acoustic Breathiness Index (ABI) para o português brasileiro.

[49] Gasparini G, Behlau M (2009). Quality of life: validation of the Brazilian version of the voice-related quality of life (V-RQOL) measure. J Voice.

[50] Behlau M, Azevedo R, Pontes P, Behlau M (2004). Voz o livro do especialista..

[51] van der Ende J, Verhulst FC, Tiemeier H (2012). Agreement of informants on emotional and behavioral problems from childhood to adulthood. Psychol Assess.

[52] Connor NP, Cohen SB, Theis SM, Thibeault SL, Heatley DG, Bless DM (2008). Attitudes of children with dysphonia. J Voice.

[53] Verduyckt I, Remacle M, Jamart J, Benderitter C, Morsomme D (2011). Voice: related complaints in the pediatric population. J Voice.

[54] Wolpert M (2014). UUses and abuses of patient reported outcome measures (PROMs): potential iatrogenic impact of PROMs implementation and how it can be mitigated. Adm Policy Ment Health.

[55] Goulart BNG, Chiari BM (2007). Testes de rastreamento x testes de diagnóstico: atualidades no contexto da atuação fonoaudiológica. Pró-Fono R Atual Cient..

[56] Behlau M, Santos LMA, Oliveira G (2011). Cross-cultural adaptation and validation of the voice handicap index into brazilian portuguese. J Voice.

[57] Teixeira LC, Rodrigues ALV, Silva AFG, Azevedo R, Gama ACC, Behlau M (2013). Escala URICA-VOZ para identificação de estágios de adesão ao tratamento de voz. CoDAS.

[58] Rodrigues G, Zambon F, Mathieson L, Behlau M (2013). Vocal tract discomfort in teachers: its relationship to self-reported voice disorders. J Voice.

[59] Ricarte A, Oliveira G, Behlau M (2013). Validação do protocolo Perfil de Participação e Atividades Vocais no Brasil. CoDAS.

[60] Moreti F, Zambon F, Oliveira G, Behlau M (2014). Cross-cultural adaptation, validation, and cutoff values of the Brazilian version of the Voice Symptom Scale-VoiSS. J Voice.

[61] Oliveira G, Hirani SP, Epstein R, Yazigi L, Behlau M (2016). Validation of the brazilian version of the Voice Disability Coping Questionnaire. J Voice.

[62] Zambon F, Moreti F, Ribeiro VV, Nanjundeswaran C, Behlau M (2022). Vocal fatigue index: validation and cut-off values of the Brazilian version. J Voice.

[63] Moreti F, Rocha C, Borrego MCM, Behlau M (2011). Desvantagem vocal no canto: análise do protocolo Índice de Desvantagem para o Canto Moderno - IDCM. Rev Soc Bras Fonoaudiol.

[64] Ávila MEB, Oliveira G, Behlau M (2010). Índice de Desvantagem Vocal no Canto Clássico (IDCC) em cantores eruditos.. Pró-Fono R Atual Cient..

[65] Ribeiro LL, Paula KMP, Behlau M (2014). Qualidade de Vida em Voz na População Pediátrica: validação da versão brasileira do Protocolo Qualidade de Vida em Voz Pediátrico. CoDAS.

[66] Santos HHANM, Aguiar AGO, Baeck E, van Borsel J (2015). Tradução e avaliação preliminar da versão em Português do Questionário de Autoavaliação Vocal para Transexuais de Homem para Mulher. CoDAS.

[67] Pernambuco L, Espelt A, Magalhães HV, Lima KC (2017). Recomendações para elaboração, tradução, adaptação transcultural e processo de validação de testes em Fonoaudiologia. CoDAS.

[68] Ribeiro LL, Verduyckt I, Behlau M (2019). Sintomas vocais na população pediátrica: validação da versão brasileira do questionário de sintomas vocais pediátrico. CoDAS.

[69] Behlau M, Oliveira G, Santos LMA, Ricarte A (2009). Validação no Brasil de protocolos de auto-avaliação do impacto de uma disfonia. Pró-Fono R Atual Cient..

[70] Beaton DE, Bombardier C, Guillemin F, Ferraz MB (2000). Guidelines for the process of cross-cultural adaptation of self-report measures. Spine.

[71] Muñiz J, Elosua P, Hambleton RK (2013). Directrices para la traducción y adaptación de los tests: segunda edición. Psicothema.

[72] AERA: American Educational Research Association. APA: American Psychological Association. NCME: National Council on Measurement in Education (2014). Standards for educational and psychological testing..

[73] Branski RC, Cukier-Blaj S, Pusic A, Cano SJ, Klassen A, Mener D (2010). Measuring quality of life in dysphonic patients: A systematic review of content development in patient-reported outcomes measures. J Voice.

[74] Bogaardt HCA, Hakkesteegt MM, Grolman W, Lindeboom R (2007). Validation of the Voice Handicap Index using Rasch analysis. J Voice.

[75] Deary IJ, Wilson JA, Carding PN, MacKenzie K, Watson R (2010). From dysphonia to dysphoria: mokken scaling shows a strong, reliable hierarchy of voice symptoms in the Voice Symptom Scale questionnaire. J Psychosom Res.

[76] Nanjundeswaran C, van Mersbergen M, Morgan K (2019). Restructuring the vocal fatigue index using mokken scaling: insights into the complex nature of vocal fatigue. J Voice.

[77] Wulff NB, Møller PR, Christensen KB, Pedersen SG, Wessel I, Dalton SO (2021). The Voice-Related Quality of Life (V-RQOL) instrument: cross-cultural translation and test of validity and reliability of the Danish version. J Voice.

[78] Pasquali L (2007). TRI – Teoria de Resposta ao Item: Teoria de procedimentos e aplicações..

[79] Andrade JM, Laros JA, Gouveia VV (2010). O uso da teoria de resposta ao item em avaliações educacionais: diretrizes para pesquisadores. Aval Psicol.

[80] Castro SMJ, Trentini C, Riboldi J (2010). Teoria da resposta ao item aplicada ao Inventário de Depressão de Beck. Rev Bras Epidemiol.

[81] Alencar SAL, Santos JP, Almeida LN, Nascimento JA, Lopes LW, Almeida AA (2022). Factorial analysis of the Brazilian version of the Vocal Tract Discomfort Scale in patients with dysphonia. J Voice.

[82] Alencar SAL (2019). A teoria de resposta ao item na avaliação de sintomas sensoriais na disfonia.

[83] Aguiar AC, Almeida LN, Pernambuco L, Palhano DB, Andrade JM, Behlau M (2020). Stages of readiness in patients with dysphonia: a proposal based on factor analysis using the URICA-V scale. J Voice.

[84] Aguiar AC, Almeida LN, Pernambuco LA, Ramos NS, Andrade JM, Behlau M (2021). Urica-VV Scale: a new research perspective of the stage of readiness for treatment in patients with dysphonia. J Voice.

[85] Almeida LN, Behlau M, Ramos NS, Barbosa IK, Almeida AA (2020). Factor analysis of the Brazilian Version of the Voice-Related Quality of Life (V-RQOL) Questionnaire. J Voice.

[86] Almeida LN, Almeida AA (2020). Autoavaliação dos sintomas vocais e estratégias de enfrentamento na disfonia: nova perspectiva com base na Teoria de Resposta ao Item..

[87] Ramos NS (2020). Validação do índice de desvantagem vocal com base na teoria de resposta ao item.

[88] Barbosa IK (2020). Validação brasileira da Escala de Controle Percebido no Presente sobre a Voz (ECPP-V) com base na teoria de resposta ao item.

[89] Pritchard MJ (2006). Making effective clinical decisions: a framework for nurse practitioners. Br J Nurs.

[90] Roy N, Barkmeier-Kraemer J, Eadie T, Sivasankar MP, Mehta D, Paul D (2013). Evidence-based clinical voice assessment: a systematic review. Am J Speech Lang Pathol.

[91] Kreiman J, Gerratt BR, Ito M (2007). When and why listeners disagree in voice quality assessment tasks. J Acoust Soc Am.

[92] Englert M, Madazio G, Gielow I, Lucero J, Behlau M (2018). Influência do fator de aprendizagem na análise perceptivo-auditiva. CoDAS.

[93] Paiva MAA, Rosa MRD, Gielow I, Silva IM, Sousa ESS, Silva ACF (2021). Auditory skills as a predictor of rater reliability in the evaluation of vocal quality. J Voice.

[94] Takishima M, Gielow I, Madazio G, Behlau M (2020). The impact of vocal tuning in the perceptual auditory judgment of normal and deviated voice qualities. CoDAS.

[95] Buder EH, Kent RD, Ball MJ (2000). Voice quality measurement..

[96] Bastian RW, Keidar A, Verdolini-Marston K (1990). Simple vocal task for detecting vocal fold swelling. J Voice.

[97] Snell EN, Plexico LW, Weaver AJ, Sandage MJ (2020). Quantifying vocal power: correlation of whole-body anaerobic power to vocal function measures. J Speech Lang Hear Res.

[98] Florencio VO, Almeida AA, Balata P, Nascimento S, Brockmann-Bauser M, Lopes LW (2021). Differences and reliability of linear and nonlinear acoustic measures as a function of vocal intensity in individuals with voice disorders. J Voice.

[99] Almeida LNA (2020). Autoavaliação dos sintomas vocais e estratégias de enfrentamento na disfonia: nova perspectiva com base na teoria de resposta ao item.

[100] Peixoto JM, Santos SMO, Faria RMD (2018). Processos de desenvolvimento do raciocínio clínico em estudantes de medicina. Rev Bras Educ Med.

