# Supplementary material for: Reducing the GAP between science and clinic: lessons from academia and professional practice - part A: perceptual-auditory judgment of vocal quality, acoustic vocal signal analysis and voice self-assessment
Source: Codas. 2022 Aug 1;34(5):e20210240. doi: 10.1590/2317-1782/20212021240en (PMC9886186; doi:10.1590/2317-1782/20212021240en)
Supplement: Translation

# Translation: Reduzindo o GAP entre a ciência e a clínica: lições da academia e da prática profissional – parte A: julgamento perceptivo-auditivo da qualidade vocal, análise acústica do sinal vocal e autoavaliação em voz

**DOI:** 10.1590/2317-1782/20212021240pt

**Authors:** Mara Behlau, Anna Alice Almeida, Geová Amorim, Patrícia Balata, Sávio Bastos, Mauricéia Cassol, Ana Carolina Constantini, Claudia Eckley, Marina Englert, Ana Cristina Cortes Gama, Ingrid Gielow, Bruno Guimarães, Livia Ribeiro Lima, Leonardo Lopes, Glaucya Madazio, Felipe Moreti, Vanessa Mouffron, Katia Nemr, Priscila Oliveira, Marina Padovani, Vanessa Veis Ribeiro, Kelly Silverio, Thays Vaiano, Rosiane Yamasaki

**Affiliations:** 1 Centro de Estudos da Voz – CEV - São Paulo (SP), Brasil.; 2 Escola Paulista de Medicina – EPM, Universidade Federal de São Paulo – UNIFESP - São Paulo (SP), Brasil.; 3 Universidade Federal da Paraíba – UFPB - João Pessoa (PB), Brasil.; 4 Universidade Federal de Alagoas – UFAL - Maceió, AL, Brasil.; 5 APTA COMUNICAÇÃO - Recife (PE), Brasil.; 6 Universidade Federal de Pernambuco – UFPE - Recife (PE), Brasil.; 7 Centro de Fotobiomodulação e Saúde – CFOTOBIOS - Belém (PA), Brasil.; 8 Universidade Federal de Ciências da Saúde de Porto Alegre – UFCSPA - Porto Alegre (RS), Brasil.; 9 Universidade Estadual de Campinas – UNICAMP - Campinas (SP), Brasil.; 10 Faculdade de Ciências Médicas da Santa Casa de São Paulo – FCMSCSP - São Paulo (SP), Brasil.; 11 Universidade Federal de Minas Gerais – UFMG - Belo Horizonte (MG), Brasil.; 12 Clínica Bruno Guimarães Serviços de Fonoaudiologia e Fisioterapia - Fortaleza (CE), Brasil.; 13 Centro Universitário da Faculdade de Medicina do ABC – FMABC - Santo André (SP), Brasil.; 14 Complexo Hospitalar Municipal de São Bernardo do Campo – CHMSBC - São Bernardo do Campo (SP), Brasil.; 15 Universidade de São Paulo – USP - São Paulo (SP), Brasil.; 16 Faculdade de Odontologia de Bauru – FOB, Universidade de São Paulo – USP - Bauru (SP), Brasil.

**Keywords:** Voz, Distúrbio da Voz, Qualidade da Voz, Acústica, Autoteste

## Abstract

No XXVIII Congresso Brasileiro da SBFa, 24 especialistas reuniram-se e, a partir de um posicionamento condutor sobre pesquisa científica como ferramenta de conexão entre laboratório e clínica, cinco frentes de conhecimento da especialidade de voz foram discutidas: 1. Julgamento perceptivo-auditivo da qualidade vocal; 2. Análise acústica do sinal vocal; 3. Autoavaliação em voz; 4. Técnicas tradicionais de terapia; 5. Técnicas modernas de eletroestimulação e fotobiomodulação em voz. A parte “a” desta publicação é a consolidação das análises dos três primeiros aspectos. A tendência no julgamento perceptivo-auditivo da qualidade vocal é o uso de protocolos padrão. A avaliação acústica do sinal vocal é acessível e pode ser feita de modo descritivo ou por extração de parâmetros, preferindo-se medidas multiparamétricas. Finalmente, a análise do próprio indivíduo fecha essa tríade de documentação fonoaudiológica, que será base para a conclusão da avaliação, referência para monitoramento do progresso e avaliação de resultado de tratamento.

## INTRODUÇÃO

A área de voz é uma das cinco especialidades originais no Brasil^(1)^, com uma produção intensa, reconhecida nacional e internacionalmente^(2)^. Enquanto o principal papel da ciência é o avanço do conhecimento, sem a obrigação de que seja aplicado clinicamente, o exercício clínico tem sob sua responsabilidade oferecer o melhor tratamento disponível ao indivíduo, com a finalidade de considerar as evidências científicas existentes. A prática clínica baseada em evidências irá fornecer classificações sobre sua qualidade e força de recomendações, com estabelecimento prioridades, sistematização e hierarquização os processos de tomada de decisão, além de considerar que os valores do paciente é uma necessidade dos profissionais da área da saúde. Contudo, nem sempre há evidências produzidas rapidamente e que representem a evolução de uma especialidade e, além disso, a adesão às diretrizes científicas pode ser variável, o que requer uma disseminação ativa e estratégias diversas de implementação na realidade clínica^(3)^. A distância entre a pesquisa e a clínica pode ser, em alguns casos, grande e incômoda.

O exercício da atividade clínica na área de voz é complexo, envolve o uso do conhecimento científico existente, que é tangível, mas também fazem parte aspectos menos mensuráveis, como a Metaterapia^(4)^, competência profissional baseada na expertise clínica e que representa o “como fazer silencioso” na reabilitação vocal^(5)^.

Os cursos de graduação, no Brasil e no exterior, focam em integrar os diversos conteúdos teóricos para a formação do fonoaudiólogo, com uma estrutura de programa, para favorecer a associação da teoria com a prática, e contam com os supervisores de estágio para direcionar boas práticas clínicas^(6)^. Contudo, não cabe à graduação formar o especialista, por diversos motivos entre os quais se destacam a carga horária e a necessidade de maturidade profissional.

De modo semelhante ao que ocorre no estágio da graduação, o atendimento clínico profissional também integra conteúdos teóricos à prática, mas espera-se que o fonoaudiólogo seja capaz de individualizar sua intervenção, não somente de acordo com o diagnóstico individual, mas considerando a personalidade do paciente e o contexto de uso da voz, particularmente em seu papel social e profissional. Na clínica tomam-se decisões para resolver o problema do paciente, sem que ele seja forçado a se encaixar em um modelo teórico pré-existente; em outras palavras, há uma maior liberdade que nos atendimentos das clínicas-escola. Há regras a serem respeitadas, mas há também uma flexibilidade consciente relacionada à experiência e competência profissionais.

Clínicos usam conhecimentos explícitos, resultados do avanço da ciência, e tácitos, decorrente da experiência e prática clínica. Questões clínicas devem ser estruturadas e analisadas com base teórica; contudo, o atendimento profissional vai além desse aspecto e pode envolver metodologias e abordagens inovadoras, ainda não totalmente testadas. O conhecimento tácito é produzido pela carga de experiência profissional, por habilidades desenvolvidas, valores no atendimento e constituem um outro tipo de evidência que tem grande impacto na tomada de decisão. O papel dos docentes é incentivar a obtenção de referenciais teóricas e de conhecimento explícito, além de favorecer o desenvolvimento de raciocínio clínico e a aquisição de habilidades práticas. O conhecimento científico estruturado não é suficiente para o desenvolvimento clínico, mas é uma base essencial para um atendimento consciente e consequente. Reconhecer dúvidas nos atendimentos faz parte da maturidade profissional e não deve afetar a integridade do fonoaudiólogo clínico; o risco que não se pode correr é de uma formação acrítica e que não estimula o pensamento reflexivo.

O objetivo do presente artigo é registrar um resumo da Sessão Científica da sala Mérito Mara Behlau, ocorrida no dia 14 de novembro de 2020, uma das atividades do XXVIII Congresso Brasileiro de Fonoaudiologia, organizado pela Sociedade Brasileira de Fonoaudiologia. O presente artigo apresenta dados sobre a metodologia científica como instrumentos de aproximação da ciência e da clínica e aponta a evolução da área de voz como ciência, apresentando, lado a lado, as bases teóricas explícitas e as tácitas da experiência profissional. Fonoaudiólogos sêniores e jovens pesquisadores, das mais reconhecidas tradições acadêmicas, desenvolveram um diálogo com clínicos experientes e respeitados pelo mercado, acerca de abordagens com variável nível de evidência, entre as quais, o julgamento perceptivo-auditivo da qualidade vocal, a avaliação acústica do sinal vocal, a autoavaliação em voz, considerando-se diversos aspectos relacionados ao problema vocal, técnicas tradicionais de terapia e, finalmente, técnicas modernas de eletroestimulação e fotobiomodulação aplicada à reabilitação vocal. Na primeira parte dessa publicação, são apresentadas considerações essenciais sobre qualidade das pesquisas científicas e oferecida uma análise comentada sobre os três primeiros aspectos debatidos nessa sessão científica, a saber: 1. Julgamento perceptivo-auditivo da qualidade vocal; 2. Análise acústica do sinal vocal; 3. Autoavaliação em voz.

## PESQUISA CIENTÍFICA: DO LABORATÓRIO À ROTINA CLÍNICA

O GAP entre a pesquisa e a clínica é histórico. As pesquisas científicas eram baseadas principalmente em opiniões de especialistas e séries de casos da prática clínica. A maioria desses estudos não possuía validade, e suas conclusões não eram generalizáveis. Dessa forma, as evidências descritas nas pesquisas científicas muitas vezes não eram aplicáveis à prática, o que distanciava a pesquisa e a clínica. Por outro lado, a prática clínica era baseada em um aprendizado adquirido na graduação, e quando havia dúvida, recorria-se aos colegas especialistas ou referências na área^(7)^. Com o passar do tempo, as evidências científicas passaram a ser cada vez de mais fácil acesso para os clínicos, com pesquisas disponíveis em periódicos de acesso aberto, cursos, congressos, livros, *lives* de conteúdo científico, entre outros. Diante do grande volume de informações disponíveis, o desafio que antes era o acesso, passa a ser a seleção da evidência de melhor qualidade e aplicabilidade clínica^(7,8)^.

Para reduzir o GAP entre a ciência e a clínica, na década de 90 surgiu um movimento denominado Prática Baseada em Evidências (PBE). A PBE consiste em associar as perspectivas do paciente e a experiência clínica, com a capacidade de analisar e aplicar de forma consciente e criteriosa a melhor evidência científica disponível^(7-9)^ (Figura 1). Esse movimento faz com que o desenvolvimento da pesquisa busque a obtenção de evidências que respondam as dúvidas da prática. A partir do uso da PBE é possível reduzir a incerteza na tomada de decisão clínica, facilitar a escolha de procedimentos diagnósticos com maior acurácia, diminuir tempo e custo do tratamento, reduzir o risco e aumentar o efeito do tratamento, dentre outros benefícios. Dessa forma é possível integrar evidências clínico-epidemiológicas com a experiência prática cotidiana de cada profissional, ou seja, oferecer cuidados ideais de acordo com as condições reais.

**Figura 1:** Composição da Prática Baseada em Evidências

Decorrente desse movimento, em 2004 foi reconhecida a Fonoaudiologia Baseada em Evidências (FBE), cujo foco é nos distúrbios da comunicação^(9)^. A implementação da FBE na prática clínica segue as seguintes etapas: identificar o problema clínico e elaborar uma pergunta de pesquisa, para a qual pode-se usar o acrônimo PICO (Paciente, Intervenção, Comparação e *Outcomes* - desfecho) ou suas variações; identificar evidências que respondam a pergunta clínica nas bases de dados; analisar criticamente a evidência (validade, confiabilidade, qualidade e aplicabilidade); implementar as conclusões científicas, como pilares para a prática clínica, considerando a experiência clínica e as especificidades e preferências do paciente^(9)^. Para auxiliar na implementação diária da FBE, a associação recomenda ainda o acrônimo DECIDE, no qual D refere-se a “Defina a pergunta”, E a “Extrapole as evidências”, C a “Considere a experiência”, I a “Incorpore as perspectivas e necessidades do paciente”, D a “Desenvolva um plano de tratamento” e E a “Examine sua decisão clínica”^(9)^.

Entretanto, a vitalidade científica na Fonoaudiologia faz com que, muitas vezes, no processo de busca, o clínico encontre um grande volume de informações, com evidências heterogêneas e qualidade variável. Diante disso, a FBE propõe uma hierarquização das pesquisas por nível de evidência, buscando respaldar a prática no melhor nível de evidência disponível. A forma tradicional de hierarquia do nível de evidência é pelo delineamento das pesquisas que considerava apenas a validade^(10)^. Porém, após essa classificação primária, é importante que as pesquisas sejam reclassificadas de acordo com a classificação da qualidade (ou certeza) das evidências e a força das recomendações^(11)^.

Diversas classificações de delineamento são encontradas na literatura, porém, optou-se aqui por utilizar uma classificação elaborada a partir da epidemiologia^(12)^, da *Clinical Trials* ^(13)^ e da ASHA^(9)^. De acordo com o delineamento, os estudos podem ser classificados em estudos com dados primários (estudos individuais cujos dados brutos são coletados pelo pesquisador) e secundários (combina as descobertas de estudos primários e fornece conclusões sobre esse corpo de evidências)^(9)^. Os estudos primários podem ser subdivididos por meio da comparabilidade em observacionais (exposição não-controlada e alocação não-aleatória), quasi-experimentais (intervenção controlada e alocação não-aleatória dos participantes nos grupos de pesquisa) e experimentais (intervenção controlada e alocação aleatória dos participantes nos grupos de pesquisa). Há ainda um subgrupo nos estudos quasi-experimentais denominado de estudos de intervenção antes e após (intervenção não-controlada, alocação não pode ser aleatória porque há apenas um grupo de intervenção). Na categoria dos observacionais encontram-se os estudos caso-controle (seleção dos grupos com base na doença, observação retrospectiva da exposição), coorte (seleção dos grupos com base em uma característica em comum e classificação com base na exposição, observação prospectiva da doença) e transversal (corte no tempo com mensuração de doença e exposição simultaneamente)^(12)^. Os estudos experimentais (ensaios clínicos randomizados) podem ser subclassificados de acordo com a fase, a partir de adaptação para estudos comportamentais, da seguinte forma: Fase I inicial – estudos laboratoriais; Fase I – análise da segurança de intervenção de efeito imediato com sujeitos normais; Fase II – análise da segurança e efeito de intervenção de efeito imediato com sujeitos doentes; Fase III – análise de segurança e eficácia de intervenção em sujeitos doentes; Fase IV – *follow up* e pós mercado^(13)^.

Para ordenar o nível de evidência de acordo com o delineamento, propõe-se uma adaptação da proposta do *Oxford Centre for Evidence-Based Medicine (OXFORD CENTRE FOR EVIDENCE-BASED MEDICINE)* e da proposta de revisão de delineamentos^(11)^ para os estudos fonoaudiológicos. Propomos que as pesquisas sejam ordenadas conforme mostra a Figura 2, que mostra a evolução da pirâmide tradicional das evidências científicas, baseada na validade dos estudos, para uma pirâmide com linhas onduladas, que destaca que os diversos tipos de delineamento não são categorias estanques para, chegarmos a uma nova proposta na qual as revisões sistemáticas são uma lente através da qual as evidências são analisadas.

**Figura 2:** Evolução da pirâmide tradicional para uma proposta na qual as revisões sistemáticas são apresentadas como uma lente através da qual as evidências são visualizadas e aplicadas

É importante ressaltar que o delineamento está diretamente relacionado a pergunta clínica que se pretende responder, podendo ser agrupados da seguinte forma: acurácia diagnóstica, prevalência e validação – estudos transversais; fator de risco ou proteção e etiologia – estudos coorte ou caso-controle; tratamento e prevenção – estudo experimental, quasi-experimental e intervenção antes e após^(9)^.

Além do delineamento, deve-se considerar a metodologia do estudo para identificar quaisquer limitações da evidência^(9)^. Para isso pode-se usar instrumentos para análise da qualidade metodológica ou risco de viés. Pesquisas secundárias com delineamento de revisão sistemática geralmente incluem avaliação da qualidade metodológica, assim como uma conclusão única, o que faz com que esse delineamento seja o que fornece o melhor subsídio científico para a tomada de decisão clínica e para o desenvolvimento de diretrizes assistenciais^(14)^.

Atualmente o ciclo da FBE é de complementaridade entre clínica e pesquisa. Ele se estabelece a partir da clínica por meio das dúvidas da prática, da pesquisa por meio do desenvolvimento de estudos que forneçam evidências para responder as dúvidas, e novamente da clínica que absorve e aplica a evidência, trazendo, a partir disso, novas dúvidas. Constitui-se assim o infinito ciclo de parceria e complementaridade desenvolvido entre a clínica e a pesquisa.

Ainda há muitos desafios para que a PBE seja implementada e possa atender as demandas práticas da clínica. O Quadro 1 mostra alguns desses desafios.

**Quadro 1:** Desafios da implementação da Prática Baseada em Evidências

**Domínio do desafio**	**Desafios**
Busca de evidências	Unitermos nacionais limitados e muito gerais
	Grande período entre o desenvolvimento, a submissão, o aceite, e período de embargo para publicação, que faz com que a disponibilidade da evidência seja atrasada em relação a velocidade da clínica
	Baixo nível de evidência de estudos para alguns temas
Seleção e comparação de evidências	Ausência de cálculo amostral, amostras pequenas e heterogêneas
	Procedimentos de padrão de referências de alterações vocais heterogêneos
	Ausência de padronização de desfecho
	Diversidade de classificações da disfonia
	Ausência de seguimento para verificar efeitos no médio e longo prazo
	Variabilidade de variáveis temporais na intervenção
Consumo de evidências	Baixo consumo de estudos pelos clínicos
	Planos de saúde nacionais não exigem o embasamento científico da prática

O grande desafio do futuro é a ciência da implementação. Somente assim será possível oferecer a melhor assistência fonoaudiológica para os pacientes dos distúrbios da comunicação.

### Julgamento perceptivo-auditivo da qualidade vocal

O julgamento perceptivo-auditivo da qualidade vocal é comumente nomeado de análise perceptivo-auditiva da voz (APA) e constitui-se no principal instrumento da avaliação vocal fonoaudiológica. Nessa forma de análise, utilizamos o julgamento perceptivo-auditivo de modo sistemático como uma decisão de resposta aos estímulos sonoros^(15-19)^. O fato de a voz ser um fenômeno perceptivo por natureza faz da análise perceptiva a melhor estratégia de avaliação dos distúrbios vocais, sendo considerada o padrão ouro da avaliação vocal. As descrições perceptivas são intuitivas e carregam importantes significados clínicos^(15-18)^.

Por meio do julgamento perceptivo-auditivo, podemos: descrever as características vocais do paciente, identificar se a voz é normal ou desviada, classificar o tipo de desvio predominante (rugosa, soprosa, tensa ou uma combinação de desvios) e a intensidade da alteração^(18)^; verificar a funcionalidade vocal com a utilização de tarefas que promovam diferentes ajustes da musculatura laríngea e perilaríngea^(20)^; auxiliar no diagnóstico diferencial das disartrias, entre elas a flácida, espástica, atáxica, hipocinética, hipercinética e as mistas^(21)^; comparar resultados de tratamentos pré e pós-intervenção, seja medicamentosa, cirúrgica ou por reabilitação vocal; e elaborar o raciocínio clínico para a compreensão do distúrbio vocal, para a tomada de decisão e para o processo de reabilitação vocal^(17,18,20-22)^.

A subjetividade envolvida nessa forma de avaliação é uma das principais críticas à análise perceptiva. Existem fatores que interferem no resultado da análise perceptiva. Alguns desses fatores envolvem erros aleatórios, difíceis de serem controlados. Outros envolvem erros sistemáticos, erros conhecidos que são mais passíveis de controle, como o treinamento auditivo do avaliador, a escala ou protocolo de avaliação, os parâmetros vocais e as tarefas de fala . O treinamento auditivo é fundamental tanto para o desenvolvimento das referências internas de avaliadores com pouca experiência como para a recalibração da percepção de avaliadores experientes^(15)^. Pode ser realizado com a utilização de vozes humanas, com diferentes tipos e graus de desvios vocais, e de vozes sintetizadas. O uso de estímulos âncoras oferece referências externas aos avaliados e calibra a percepção auditiva^(19,23)^.

Existem diferentes protocolos padronizados para a análise perceptiva. A escala japonesa GRBAS^(24)^ e o Protocolo CAPE-V^(25)^ são amplamente utilizados para fins clínicos e científicos. Ambos contêm parâmetros vocais de confiabilidade elevada, como o G - grau geral de desvio vocal, a rugosidade e a soprosidade^(17,23,26)^. Enquanto a GRBAS usa uma escala numérica de 4 pontos, o protocolo CAPE-V usa a escala analógico-visual de 100mm, o que oferece maior precisão na análise, permitindo uma apreciação mais acurada dos progressos vocais em terapia, mesmo que de menor magnitude. Os dois protocolos apresentam um número limitado de parâmetros a serem avaliados, o que reflete a tendência atual da literatura; porém, o CAPE-V prevê o registro de tipos de vozes menos comuns na clínica vocal.

A escolha das tarefas de fala é um dos pontos chaves da APA. Existem tarefas de fala menos específicas, como emissão de vogal sustentada, contagem de números, conversa espontânea e emissão de voz cantada, como “parabéns a você”; e mais específicas, como as tarefas de variação de frequência (habitual, agudo, grave e glissando) e de intensidade (habitual, fraco e forte) do campo dinâmico vocal - CDV, que proporcionam informações sobre a funcionalidade da musculatura laríngea e perilaríngea^(20)^; o uso de frases com motivação fonética, como as do CAPE-V, auxilia na diferenciação das disfonias neurológicas das disfonias comportamentais; e as provas de diadococinesia laríngea e fonoarticulatória, tornam-se importantes nas disfonias por alteração do controle motor^(21,27)^. Vogais geralmente evidenciam características da fonte do som e as amostras de fala encadeadas permitem uma avaliação mais abrangente do uso da voz, com aspectos que podem facilmente ser minimizados se forem consideradas apenas emissões sustentadas de vogais.

Além dos fatores de interferência na APA já descritos, estudos mostram que vieses cognitivos, como o conhecimento da história clínica, da informação diagnóstica e do contexto clínico podem influenciar a APA^(28-30)^. Dessa forma, pesquisadores e clínicos devem estar atentos às possibilidades de categorização automática disparadas pelo conhecimento disponível e experiência prévia na realização da APA.

### Avaliação acústica do sinal vocal

Outro importante instrumento da avaliação vocal, que aliado ao julgamento perceptivo-auditivo é de grande valor clínico é a avaliação acústica do sinal vocal. Essa análise, considerada menos subjetiva que o julgamento perceptivo-auditivo tem como principais objetivos: documentar a qualidade vocal, quantificar diversos aspectos da produção da voz, detectar distúrbios vocais e/ou laríngeos, realizar triagens, acompanhar o processo terapêutico e registrar a voz do paciente^(31,32)^. Além disso, ela desempenha um importante papel em ajudar o avaliador a ver o que está ouvindo^(33,34)^.

Historicamente, a avaliação acústica era restrita a grandes laboratórios e centros de pesquisa universitários pela necessidade de uso de computadores de elevado custo (minicomputadores) e com alta capacidade de processamento^(31,32)^. A análise era essencialmente feita na vogal sustentada, por variar menos que a fala encadeada possibilitando uma extração de parâmetros mais fácil^(35-37)^. Encontrava-se uma grande dificuldade em analisar vozes com grandes desvios vocais devido à necessidade de identificar os limites individuais de cada ciclo, o que é menos evidente em vozes muito^(38)^. Nas últimas décadas os avanços tecnológicos permitiram que a avaliação acústica fosse utilizada em diversos *softwares* de computadores pessoais, de uso gratuito (como o Praat, de Paul Boersma e David Weenink, *Institute of Phonetic Sciences*, Amsterdam) ou de baixo custo (como o VoxMetria e FonoView, CTS Informatica, Brasil) aumentado seu uso e assim, sua crítica e reflexão^(39)^.

A avaliação acústica, embora frequentemente associada à extração de parâmetros, pode também ser descritiva, baseada essencialmente na espectrografia, uma análise qualitativa do sinal vocal que permite a avaliação de quaisquer tipos de sinais vocais, até mesmo de vozes com desvios extremos, tanto de amostras de vogal sustentada como da fala^(40,41)^. A análise da espectrografia acústica da voz exige treinamento do avaliador e é mais difícil de ser padronizada uma vez que a análise do traçado é um julgamento perceptivo-visual com boa dose de subjetividade. No entanto, pesquisas têm sido realizadas com o objetivo de desenvolver um protocolo padrão para essa análise^(42-44)^ que tanto contribui para a clínica ao fornecer um *feedback* visual imediato para o clínico e para o paciente.

Por sua vez, a avaliação por parâmetro acústico é fácil de ser padronizada, uma vez que a análise fornece um número. Tradicionalmente a análise paramétrica é feita por medidas isoladas, tais como: frequência fundamental, uma medida robusta; medidas de perturbação no curto-prazo, como *jitter* e *shimmer*; medidas de ruído, como *signal to noise ratio* – SNR, *harmonic to noise ratio -* HNR, *glottal to noise excitation –* GNE, entre outras. Vale ressaltar que as pesquisas buscam relacionar medidas acústicas com a qualidade vocal percebida auditivamente e a intensidade da disfonia^(36)^, mas frequentemente as alterações em parâmetros acústicos isolados não refletem o que o clínico ouve. Tais parâmetros em geral consideram a vogal sustentada como tarefa para análise, o que torna a avaliação menos assertiva e menos representativa da voz como um todo^(37)^. Dessa forma, há uma busca por medidas que sejam mais confiáveis e que considerem também a fala encadeada, como o *cepstral peak prominence* - CPP^(37,45,46)^. O uso de medidas multiparamétricas, recentemente introduzidas na literatura, tem-se tornado muito popular, tais como o *Acoustic Voice Quality Index*, AVQI^(37)^ e o *Acoustic Breathiness Index*, ABI^(16)^, ambos validados para o português brasileiro^(47,48)^ e extraídos por um *script* do programa Praat, de distribuição gratuita. Possivelmente, um dos principais desafios para o uso dessas medidas na rotina clínica é a falta de treinamento no uso dos programas que as extraem, já que o Praat não é tão amigável para uso na clínica vocal.

O julgamento perceptivo auditivo e a avaliação acústica podem ser considerados duas faces de uma mesma moeda e são a base da avaliação vocal na perspectiva do clínico. Ambas devem ser realizadas com muito critério, exigem treinamento por parte dos avaliadores e a seleção de parâmetros confiáveis. Enquanto o julgamento perceptivo-auditivo favorece a descrição da impressão geral da qualidade vocal, a avaliação acústica permite identificar aspectos essenciais da contribuição da fonte e dos filtros de ressonância na produção vocal.

### Protocolos de autoavaliação em voz

A autoavaliação na clínica de voz brasileira começou há um pouco de mais uma década com a validação do protocolo Qualidade de Vida em Voz^(49)^. A voz é biologicamente definida e socialmente moldada^(50)^. Ela apresenta uma característica multidimensional que requer a análise por diferentes perspectivas durante o processo diagnóstico de uma disfonia. O clínico tem a *expertise*, mas é o paciente quem tem a vivência da alteração vocal, um mesmo problema ao ser avaliado por diferentes perspectivas nem sempre apresentará concordância entre os avaliadores. Informantes que relatam diferentes percepções propiciam mais informações do que avaliadores que concordam completamente entre si^(51)^.

A autoavaliação auxilia na compreensão das dificuldades vividas pelo paciente^(52,53)^. É um importante recurso para o monitoramento clínico e pode auxiliar no diagnóstico^(54)^. Existem instrumentos de autoavaliação que ajudarão tanto no rastreio quanto no diagnóstico, em ambos os casos acurácia e precisão são fundamentais^(55)^. Para rastear uma alteração vocal, o instrumento deve ter alta sensibilidade, identificando as pessoas que apresentam uma alteração na voz ou fator de risco para a disfonia. Já para o diagnóstico, o instrumento deve ter alta especificidade, pois ele não pode captar outras alterações/problemas além da disfonia.

Atualmente, no Brasil, tem-se disponível na clínica de voz os seguintes protocolos: Qualidade de Vida em Voz (QVV)^(49)^, Índice de Desvantagem Vocal (IDV)^(56)^, Escala URICA-VOZ (URICA-VOZ)^(57)^, Escala de Desconforto de Trato Vocal (EDTV)^(58)^, Protocolo de Participação e Atividades Vocais (PPAV)^(59)^, Escala de Sintomas Vocais (ESV)^(60)^, Protocolo de Estratégias de Enfrentamento das Disfonias (PEED)^(61)^ e Índice de Fadiga Vocal (IFV)^(62)^. Além de outros para populações mais específicas, como Índice de Desvantagem no Canto Moderno (IDCM)^(63)^, Índice de Desvantagem no Canto Clássico (IDCC)^(64)^, Qualidade de Vida em Voz Pediátrico (QVV-P)^(65)^, Questionário de Autoavaliação Vocal para Transexuais de Homem para Mulher (TVQ^MtF^)^(66)^, Rastreamento de Alterações Vocais em Idosos (RAVI)^(67)^ e Questionário de Sintomas Vocais Pediátrico (QSV-P)^(68)^, entre outros.

Por meio da aplicação dos protocolos é possível mensurar o impacto da doença em suas relações sociais, pessoais e profissionais, diferenciar pacientes ou agrupá-los, prognosticar resultados individuais, monitorar a evolução do paciente, avaliar a efetividade da terapia, priorizar problemas no processo de intervenção além de ajudar na tomada de decisão para a alta terapêutica^(60,65,69)^. O clínico deve então, identificar o que ele quer mapear para escolher o(s) protocolo(s) mais adequado(s) para o seu paciente.

É importante que o fonoaudiólogo priorize, em sua rotina clínica, o uso de instrumentos validados em pesquisas científicas. A validação é um processo de avaliação que utiliza testes cujas interpretações dos resultados sejam válidas (mensuração daquilo que propõe), confiáveis/precisas (reprodutibilidade teste-reteste) e equitativas (imparcialidade na informação)^(67)^.

Há diversas diretrizes para desenvolver os processos de validação de instrumentos, tradicionalmente realizados por diversas áreas, com destaque para a Educação e a Psicologia^(67,70-72)^. Por isso, é fundamental que o pesquisador escolha uma linha de validação e siga rigorosamente todas as etapas preconizadas pelos autores de modo a mensurar: 1. Evidências de validade que pode ser feita baseada em conteúdo do teste, processos de resposta, consistência interna e relação com outras variáveis; 2. Confiabilidade/ Precisão; 3. Equidade do teste; 4. Acurácia; e 5. Evidência de validade baseada nas consequências do teste^(67,72)^.

Além das etapas de evidências de validade, o pesquisador deverá escolher a corrente filosófica que irá nortear a validação do teste. Nessa perspectiva, destaca-se uma revisão que avaliou nove instrumentos de autoavaliação que são relativos ao construto de qualidade de vida relacionados à voz^(73)^. Após uma análise criteriosa, os autores referiram que os questionários abordados e usuais na área de voz apresentaram problemas no desenvolvimento e déficits em algumas de suas propriedades psicométricas. Finalizaram com a sugestão da aplicação de métodos mais contemporâneos para validação e avaliação das medidas psicométricas.

Todos os instrumentos de voz do Brasil foram validados a partir da Teoria Clássica dos Testes (TCT), mas, em âmbito internacional, já existem esforços no desenvolvimento de estudos de validação de questionários com base em modelos mais contemporâneos, como a Teoria de Resposta ao Item (TRI)^(74-77)^. Dentro dessa perspectiva, vale ressaltar brevemente as diferenças entre essas teorias.

A TCT preconiza uma estrutura em que a análise depende da amostra recrutada; atribui um valor único para os itens, com isso se obtém um escore total a partir do somatório simples dos itens; a mesma quantidade de questões assinaladas (que podem ser diferentes), há o mesmo valor de escore; e, consequentemente, não diferencia os respondentes em relação à aptidão ao atributo. A TRI busca suprir as lacunas apresentadas pela TCT, uma vez que sua generalização independe do teste e da amostra; o item é estabelecido como uma unidade básica, por isso, cada um terá o seu peso cuja influência em relação ao traço latente, decorre de seus parâmetros de discriminação e dificuldade. Assim, os instrumentos validados a partir da TRI diferenciam os indivíduos de acordo com o grau de aptidão/comprometimento testado, com a finalidade de melhorar a acurácia do instrumento. Contudo, é necessária uma amostra maior, com mais variabilidade da severidade do problema, além de modelos estatísticos e *softwares* mais sofisticados^(78-80)^.

O Brasil formou um grupo interdisciplinar e multicêntrico para o desenvolvimento das etapas de evidências de validade de protocolos de autoavaliação na área de voz, com a utilização da TRI. Até o momento, há a validação de sete instrumentos, a saber: Escala de Desconforto do Trato Vocal^(81,82)^; URICA-Voz Validada (URICA-VV)^(83,84)^; Protocolo de Qualidade de Vida em Voz (QVV)^(85)^; Escala de Sintomas Vocais (ESV)^(86)^, Protocolo de Estratégias de Enfrentamento das Disfonias^(86)^, Índice de Desvantagem Vocal^(87)^e Escala de Controle Percebido no Presente sobre a Voz (CPP-V)^(88)^.

A maioria desses protocolos, validados a partir da TRI, enfatizaram a dificuldade que os pacientes tinham em entender a diferença entre intensidade e frequência, além de responder as escalas (tipo) Likert; todos tiveram os itens com parâmetros calibrados de acordo com seu nível de informação; as medidas de sensibilidade, especificidade e acurácia dos instrumentos foram satisfatórias; além de todos instrumentos citados têm nova metodologia de cálculo, baseado nos parâmetros da TRI, com possibilidade de novo ponto de corte. Dessa forma, todos esses novos protocolos seguiram a tendência contemporânea na simplificação dos instrumentos na área de Psicometria, seja com a redução de itens, estrutura uni fatorial e/ou respostas dicotômicas; com a possibilidade de classificação de sujeitos com indicativo a apresentar risco vocal.

Esses esforços coletivos para produção de novos conhecimentos científicos puderam refletir na prática clínica, para um maior entendimento da gênese e processos que circundam a disfonia, como: instrumentos que detectam disfônicos mesmo em estágio inicial, o que mais impacta na qualidade de vida são as limitações de vida diária voltadas a questões físicas e sintomas físicos mais do que emocionais; e os sintomas podem dar indícios de ter ou não lesão fonotraumática. Por fim, deve-se conhecer mais sobre os aspectos cognitivos e comportamentais do paciente para lançar mão de estratégias de enfrentamento, controle percebido no presente e o estágio de prontidão em que o paciente se encontra para que ele se torne mais ativo e que o terapeuta seja mais assertivo na reabilitação vocal. Assim, acredita-se que os avanços nas pesquisas científicas com o uso da TRI na validação dos instrumentos de autoavaliação vocal irão facilitar a rotina clínica do fonoaudiólogo.

## COMENTÁRIOS

A qualidade vocal é um aspecto essencial na avaliação de um paciente com queixa vocal, seja no contexto de triagem, confirmação diagnóstica ou monitoramento de tratamento de um problema vocal. O julgamento perceptivo-auditivo e a avaliação acústica são as principais ferramentas clínicas para caracterizar e classificar a qualidade vocal. As duas modalidades de avaliação devem ser vistas como estratégias interdependentes e devem ser interpretadas de forma integrada à história clínica do problema de voz, à autoavaliação de aspectos relacionados ao problema de voz na vida do indivíduo e ao exame médico laríngeo.

O fonoaudiólogo deve ser capaz de coletar informações auditivas e acústicas do paciente, sintetizá-las, interpretá-las e tomar uma decisão acerca da intervenção que deve ser realizada ou emitir julgamento da efetividade de uma abordagem já implementada^(89)^. Um dos grandes desafios na área de voz é compreender o papel do julgamento perceptivo-auditivo e das medidas obtidas na avaliação acústica no processo de tomada de decisão. Embora, tradicionalmente, haja um consenso entre clínicos de que a avaliação vocal seja multidimensional, não há evidências científicas que suportem essa afirmação^(90)^.

Especificamente quanto ao julgamento perceptivo-auditivo, um dos desafios é melhorar a confiabilidade e a acurácia na caracterização da qualidade vocal. De maneira geral, ouvintes com experiências auditivas semelhantes ou que tiveram treinamento auditivo durante sua formação apresentam melhor confiabilidade inter e intra indivíduos^(34)^. Dessa forma, dois pontos importantes devem ser considerados: o treinamento prévio dos juízes para melhorar a confiabilidade e acurácia no julgamento perceptivo-auditivo e o estabelecimento de especificações básicas sobre esse treinamento para, efetivamente, melhorar o desempenho nas análises realizadas^(15,91,92)^. O treinamento deve envolver habilidades relacionadas ao processamento auditivo central e aspectos específicos relacionados à tarefa de percepção. Juízes iniciantes demonstraram menor confiabilidade no julgamento do grau geral do desvio vocal quando apresentavam limitações nas habilidades de resolução temporal e interação binaural^(93)^. Um outro estudo com ouvintes afinados e desafinados demonstrou que afinação vocal do juiz não é um pré-requisito para a realização de uma boa avaliação perceptivo-auditiva da voz. Por sua vez, os participantes com dificuldade no processamento de padrões temporais apresentaram menor confiabilidade intra sujeito no julgamento perceptivo-auditivo, independentemente de serem ou não afinados^(94)^. Tal achado reforça a importância do treinamento de habilidades auditivas em programas de desenvolvimento da habilidade de realizar julgamento perceptivo-auditiva da qualidade vocal.

Por sua vez, a maior contribuição da análise acústica é viabilizar a documentação do sinal vocal, o monitoramento desse sinal ao longo da reabilitação ou aperfeiçoamento vocal, e a possibilidade de quantificar o desvio percebido auditivamente e caracterizá-lo visualmente na espectrografia. Existem 15 modelos referidos na literatura para caracterização acústica da voz^(95)^ e inúmeras medidas que podem ser obtidas a partir desses modelos. Nesse sentido, o clínico precisa decidir quais medidas mais efetivas para finalidades específicas no contexto clínico.

A busca por medidas acústicas que representem determinados descritores de qualidade vocal, como rugosidade e soprosidade, é um antigo desafio para a comunidade científica. A grande quantidade de medidas acústicas disponíveis com o objetivo de correlacionar os achados acústicos com os perceptivo-auditivos foi evidenciada em uma metanálise que avaliou medidas acústicas utilizadas para avaliação da vogal sustentada e da fala encadeada^(16)^. Os autores encontraram 85 medidas acústicas para soprosidade e 86 medidas para soprosidade. Como a metanálise é um tipo de revisão de literatura no qual diferentes estudos são comparados a partir de métodos estatísticos que reduzem viés das diferenças metodológicas entre eles, este tipo de estudo tem elevado nível de evidência. Após a análise dos estudos selecionados, os autores identificaram 12 medidas com potencial para correlação com soprosidade e 14 para a rugosidade. Um aspecto a ser destacado é que a escolha de medidas acústicas a serem utilizadas pelo clínico ou pesquisador deve se pautar no objetivo da análise pretendida.

A discriminação entre vozes normais e desviadas por meio de medidas acústicas é outro desafio para os pesquisadores da área e por isso, foi objetivo de importante estudo que analisou um banco de dados com 482 vozes^(41)^. Diferentes medidas foram comparadas individualmente e em combinação para avaliar a performance em discriminar as vozes. O diferencial deste estudo está na utilização de medidas tradicionais, cepstrais, não-lineares e medidas de quantificação de recorrência. Os resultados indicaram que *cepstral peak prominence-smoothed* (CPPS) é a medida acústica com melhor acurácia para discriminar vozes normais de alteradas e que medidas combinadas (tradicionais, como o *glottal to noise excitation* GNE) e de quantificação de recorrência (*entropy -* ENTR) podem ter resultados promissores na discriminação de vozes. Diante disto, o CPPS se firma como medida mais robusta, tanto para verificar a correlação com a soprosidade^(16,41)^ quanto para a discriminação de vozes normais e alteradas.

Um aspecto importante a ser destacado na avaliação acústica e no julgamento perceptivo-auditivo são as tarefas de fala utilizadas. Elas devem possibilitar o acesso à funcionalidade vocal em termos de variação de frequência (grave, agudo e a alternância entre eles), intensidade (fraco, habitual e forte)^(96)^, assim como resistência (emissão em tempo máximo de fonação em diferentes intensidades e frequências) e eficiência laríngea (diadococinesia laríngea)^(97)^. Além disso, o nível de pressão sonora (NPS) deve ser uma variável controlada e registrada no momento de coleta da voz. Reconhecidamente, vozes produzidas com NPS abaixo de 70 ou acima de 80dB em ambiente clínico tratado acusticamente podem distorcer a percepção de desvio vocal, sub ou superestimando as características acústicas e perceptuais da voz^(98)^.

Dessa forma, a recomendação é que a avaliação perceptivo-auditiva e acústica da qualidade vocal envolva material de fala controlado e, pelo menos em casos desafiadores, tarefas de emissão da vogal sustentada em intensidade fraca, confortável e forte, uma vez que as diferenças quantitativas e qualitativas dessas emissões podem contribuir para compreender a dinâmica laríngea, principalmente entre as condições de hiperfuncionamento vocal com ou sem lesão tecidual^(96)^. Tarefas de fala encadeada também podem ser avaliadas em diversas condições de emissão.

Se por um lado o julgamento perceptivo-auditivo e a avaliação acústica são as principais ferramentas da semiologia na perspectiva do clínico, a autoavaliação do indivíduo com problema de voz, desenvolvida com mais intensidade nas últimas duas décadas, trouxe grandes avanços na compreensão abrangente do que significa viver com uma disfonia. A adição da perspectiva do paciente em sua avaliação faz parte obrigatória da rotina da clínica vocal. Existe uma gama enorme de protocolos de autoavaliação em voz já validados no Brasil, com diferentes perspectivas de eixos de análise, tais como qualidade de vida^(49,56,59,65)^ fisiopatologia e presença de sintomas^(18,59,62,68)^, além de comportamento e cognição^(57,61)^. A dúvida que sempre surge aos clínicos, principalmente aos iniciantes é: “qual protocolo de autoavaliação vocal devo utilizar com meu paciente?” Por isso é importante o raciocínio clínico com a reflexão da queixa vocal, dados da anamnese e tomada de decisão sobre qual eixo deseja avaliar o paciente, podendo-se escolher mais de um questionário.

A validação de protocolos de autoavaliação em voz no Brasil pela teoria clássica do teste - TCT perdurou por mais de duas décadas, iniciando-se com o Questionário de Vida em Voz - QVV^(49)^. Naquele momento surgiu uma importante linha de pesquisa de validação de protocolos de autoavaliação em voz no Brasil, com estudos realizados em diversos níveis acadêmicos, de especialização a doutorado. Publicação recente reuniu recomendações para validação de testes em Fonoaudiologia^(67)^, um dos artigos mais citados da revista CoDAS no quadriênio 2017-2020, mostrando o interesse do fonoaudiólogo na área de validação, principalmente porque protocolos validados dão maior segurança ao clínico em sua tomada de decisão.

Após esses avanços iniciais, emergiu a necessidade da realização de novos estudos contemplando os instrumentos de autoavaliação como objeto de pesquisas, no intuito de assegurar que esses possam se constituir ferramentas cada vez mais eficientes para refletir de forma segura as preocupações apresentadas pelo paciente^(73)^. Linhas de pesquisas mais recentes têm-se dedicado a investigar de forma mais profunda a estrutura psicométrica dos instrumentos de autoavaliação vocal, visando a compreender melhor suas potencialidades e deficiências, além de reforçar ainda mais sua validade e confiabilidade. Estudos preliminares nessa direção^(84,85,99)^ apontaram capacidade de discriminação diferenciada entre os itens de um mesmo instrumento, conduzindo à constatação que tais itens devem ser considerados de forma diferenciada na obtenção do escore total dos instrumentos e em sua interpretação.

Tais reflexões culminaram na aplicação de teorias mais contemporâneas de validação para esses instrumentos, como a Teoria de Resposta ao Item (TRI), que tem o objetivo de solucionar tais deficiências, atribuindo pesos diferenciados a cada item investigado de acordo com sua influência em relação ao parâmetro investigado. O avanço dessas pesquisas já permitiu a obtenção de novas versões atualizadas e psicometricamente mais robustas de diversos instrumentos de autoavaliação vocal, que trazem implicações diretas na melhor qualidade da informação obtida por esses instrumentos, em âmbito clínico e acadêmico. Contudo, pelo fato de os escores resultantes não serem facilmente obtidos e nem interpretados clinicamente, seu uso ainda não está disseminado na rotina clínica.

O processo ensino-aprendizagem do uso de julgamentos perceptivos, avaliação acústica e análise de protocolos de autoavaliação em voz só se fará de modo adequado se for inserido em um programa de raciocínio clínico desde o início da formação acadêmica do fonoaudiólogo. Os dados obtidos devem ter significado e devem fazer parte da tomada de decisão clínica. O raciocínio clínico, que tem como base uma ótima anamnese e uma avaliação precisa do paciente, é uma habilidade cognitiva a ser desenvolvida por experiência ou treinamento^(100)^. Desenvolver o raciocínio clínico deve ser preocupação central na formação dos jovens fonoaudiólogos e pode ser promovido por uma abordagem teórica e estruturada, associada à observação de clínicos experientes em atendimentos nas universidades. Esse processo empodera o profissional, confere autonomia e promove habilidades essenciais para um trabalho interdependente na área de saúde.

## CONCLUSÃO

A área de voz é ágil e tem apresentado uma renovação constante e intensa nas duas últimas décadas. Funcionalidade vocal e decisão clínica consciente devem ser o processo de posicionamento do fonoaudiólogo frente ao paciente. A análise perceptivo-auditiva tem importância fundamental como “porta-voz da voz”, traduzindo em termos perceptivos a identidade vocal do indivíduo. A análise acústica, aliada à auditiva, é a mais fiel documentação vocal, permitindo a descrição da contribuição da fonte sonora e dos filtros de ressonância, além de permitir o registro de todo o estilo de comunicação do indivíduo. O clínico tem a expertise na área de voz, mas é o paciente quem vive com o problema de voz e somente ele, por meio dos protocolos de autoavaliação, poderá traduzir essa vivência em impactos nas dimensões familiar, social, profissional e emocional da vida. A ciência simplificou essa avaliação clínica da percepção do paciente, com a revalidação dos protocolos de autoavaliação do impacto do problema de voz nos diversos aspectos da vida; compreende-se hoje que os itens investigados têm relevância diversa e o conhecimento que essa informação já vem trazendo permitirá a elaboração de programas de atenção e prevenção dos distúrbios da voz baseados em dados científicos. São essas três análises: auditiva, acústica e autoavaliação do impacto dos distúrbios que formam a documentação essencial do paciente e permitem a avaliação dos resultados de um tratamento.
